# Multifunctional armored nanoemulsion of elemene combining ferroptosis induction and gut homeostasis restoration in colorectal cancer therapy

**DOI:** 10.1016/j.ijpx.2026.100517

**Published:** 2026-03-17

**Authors:** Qianyun Zhu, Huiru Li, Wenjie Lu, Dan Su, Lingzhen Ding, Jinguang Ouyang, Wenyou Fang, Tianming Wang, Shengqi Chen, Xia Liu, Song Gao, Shengyong Luo, Rongfeng Hu

**Affiliations:** a School of Pharmacy, Anhui University of Chinese Medicine, Hefei 230012, China; b Anhui Institute of Medicine (Anhui Academy of Medical Sciences), Hefei 230061, China; c School of Pharmaceutical Sciences, Tsinghua University, Beijing 100084, China; d Department of Pharmacy, The First Affiliated Hospital of University of Science and Technology of China, Hefei, Anhui 230001, China; e Anhui Zhengyao Pharmaceutical Technology Co., Ltd. Hefei, Anhui 230038, China; f Department of Gastroenterology, The First Affiliated Hospital of Anhui University of Chinese Medicine, Hefei, Anhui 230001, China; g Anhui Province Key Laboratory of Pharmaceutical Technology and Application; Key Laboratory of Xin'an Medicine, the Ministry of Education, Anhui University of Chinese Medicine, Hefei, Anhui 230038, China; h Anhui Province Joint Key Laboratory of Functional Activity and Resource Utilization of Edible and Medicinal Mushroom, Plant active peptide function food innovative manufacturing industry innovation team, Hefei, Anhui 230038, China

**Keywords:** Colorectal cancer, Armored nanoemulsion, Polysaccharide, Ferroptosis, Gut homeostasis, Synergistic therapy

## Abstract

Cancer remains a leading cause of death worldwide. Colorectal cancer (CRC) is the most common type of gastrointestinal malignancy, with the combined effect of multiple etiological factors. Multifunctional nanocomposites present a promising platform for synergistic therapy of CRC. Elemene (EL), an active component in traditional Chinese medicine, exhibits both antitumor and immunostimulatory activities. However, its clinical application is limited by poor stability, low tumor accumulation, and the difficulty of effectively harnessing its dual activities. Herein, we developed an orally administered, colon-targeted nanoemulsion, LMP@EL-CNE, comprising EL cationic nanoemulsions (EL-CNE) armored with a low-methoxyl pectin (LMP) polysaccharide shell. This system exhibits dual responsiveness to colonic microflora enzymes and pH, allowing for prolonged retention and targeted drug release in the colon. Subsequently, it generates ultra-small EL nanodroplets (∼20 nm), which enhance tumor penetration and accumulation. Notably, LMP@EL-CNE induces mitochondrial dysfunction and downregulates glutathione peroxidase 4 (GPX4), a key regulator of ferroptosis. This disruption of the balance unlocks a more potent ferroptosis response, amplifying EL's intrinsic activity in CT26 cells. In an *in vivo* orthotopic CRC model, LMP@EL-CNE exhibited potent anti-tumor activity and activated anti-tumor immunity, manifesting as a “hot” tumor microenvironment, outperforming the EL commercial preparation. Furthermore, this polysaccharide-armored nanoemulsion has been proven to restore gut homeostasis by repairing damaged epithelial barriers and reshaping the gut microbiota. In conclusion, we propose a novel nano-platform that integrates multiple functions to induce ferroptosis, activate anti-tumor immunity, and restore gut homeostasis, providing a comprehensive and effective strategy for colorectal cancer treatment.

**Chemical compounds studied in this article:**

Elemene (EL CAS No. 33880-83-0) the primary active pharmaceutical ingredient (API) with antitumor activity Polyoxyethylene hydrogenated castor oil (RH40, CAS No. 61788–85-0), a key non-ionic surfactant for nanoemulsion stabilization.

Hexadecyl trimethyl ammonium bromide (CTAB, CAS No. 57–09-0), a cationic surfactant used in the nanoemulsion formulation.

Low-methoxyl pectin (LMP, CAS No. 9000-69-5), an anionic polysaccharide constituting the functional “armor” of the nanoemulsion.

Liproxstatin-1 (Lip-1, CAS No. 950455–15-9), a potent ferroptosis inhibitor used for mechanistic validation.

Glutathione (GSH, reduced form, CAS No. 70–18-8), a central antioxidant, quantified to assess the status of the GPX4-mediated ferroptosis defense system.

Malondialdehyde (MDA, CAS No. 542–78-9), the primary end-product of lipid peroxidation, quantified as a key indicator of ferroptosis.

## Graphical abstract

**Figure:**
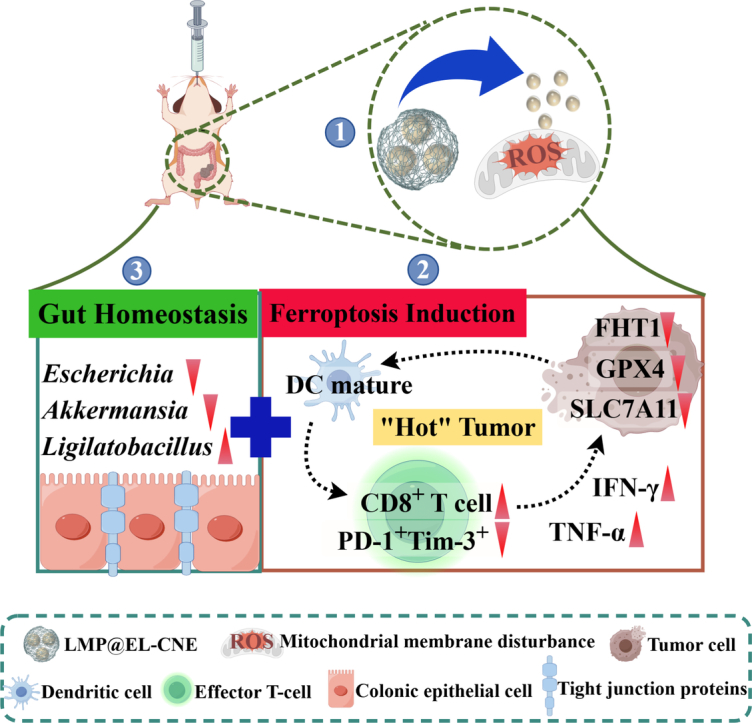

## 1 Introduction

Colorectal cancer (CRC) is the third most frequent neoplasm in the world, with almost two million cases every year, and the second leading cause of cancer-related death (Ayala-de Miguel et al., 2024; Sung et al., 2021). Many therapeutic strategies, such as surgical (Paty and Garcia-Aguilar, 2022), small molecule drug therapy (Yang et al., 2024), immunotherapy (Feng et al., 2021), and gut microbiota intervention (Pothuraju et al., 2021), have been developed, and therapeutic responses with the help of combination therapies can be improved in terms of magnitude and probability; some are promising in CRC clinical trials. However, combination therapy of multiple drugs often carries the risk of adverse reactions or even toxicity (Xu et al., 2025). The combination of KRAS G12C inhibitors with anti-EGFR therapy doubles the incidence of grade ≥ 3 adverse events compared to monotherapy (32.8% *vs.* 16.5%) and introduces new, mechanism-related toxicities (Akkus et al., 2025). Furthermore, the combination of the cytotoxic drug FTD-TPI with bevacizumab markedly exacerbates bone marrow suppression, leading to a higher incidence of neutropenia (any grade: 62.2% *vs.* 51.2%) (Prager et al., 2023). An alternative and promising strategy is the use of a delivery device to encapsulate anti-tumor drugs. This “drug-device” combination unlocks the drug's inherent multi-target anti-tumor potential through controlled release and contributes to host homeostasis, thereby enhancing both the efficacy and safety of CRC treatment (Li et al., 2025a).

Elemene (EL) is a natural sesquiterpenoid isolated from the dried rhizome of *Curcuma wenyujin* Y. H. Chen et al. (Zhai et al., 2019). It is widely used in the adjuvant treatment of malignant serous cavity effusions and gastrointestinal solid tumors (Chen et al., 2020) and has important clinical applications in cancer treatment (Chen et al., 2022; Li et al., 2021; Xie et al., 2020). Research indicates that EL exerts anti-tumor effects through multiple targets and pathways. Structurally, its small-molecule hydrophobic skeleton can disrupt the integrity of cellular and organelle membranes, potentially compromising mitochondrial electron transport and promoting the accumulation of reactive oxygen species (ROS) (Shen et al., 2025). Concurrently, the exocyclic methylene moiety of this compound acts as an electrophile, enabling irreversible covalent modification of various crucial biomolecules. It has been demonstrated that EL specifically inhibits the GPX4-mediated antioxidant defense system, thereby inducing ferroptosis in tumor cells (Zhao et al., 2024). Ferroptosis, as a potential cancer immunotherapy strategy, has a powerful anti-cancer effect and can exert chemo- and immune- multimodal anti-cancer actions (Lei et al., 2024; Yang et al., 2025). We therefore hypothesized that the ferroptosis-inducing capability of EL could be amplified by a rationally designed formulation to achieve multimodal therapy. Based on our previous research, an EL cationic nanoemulsion (EL-CNE) was engineered for tumor cell targeting, driven by the elevated electronegativity of the cancer cell plasma membrane, enhancing accumulation in tumor tissue. Subsequent induction of mitochondrial damage was achieved by exploiting the even higher intrinsic potential (approximately −150 to −200 mV) of the organelles, which combination with the downregulation of EL antioxidant defenses to amplify ferroptosis. (Nagaraj, 2025; Xiao et al., 2025; Zhang et al., 2025).

An oral colon-targeted drug delivery system (OCDDS) delivers drugs accurately to the colon *via* the oral route, achieving localized, high-concentration drug therapy and potentially modulating gut homeostasis (García-Alfonso et al., 2021; Wang et al., 2023). Low-methoxy-pectin (LMP) is a natural polysaccharide that resists digestive enzymes and reaches the colon intact (Wang et al., 2022b), where it is fermented by the gut microbiota (Raghav et al., 2023). Ongoing research provides evidence of the health benefits of LMP in the prevention and prognosis of CRC (Prajapati et al., 2025). Moreover, the galacturonic acid residues of LMP facilitate charge-mediated adsorption onto EL-CNE, as an OCDDS of polysaccharide-armored nanoemulsion, effectively preventing oral emulsion aggregation and precipitation in the gastrointestinal tract (GIT), enhancing systemic retention. While this allows for responsive release of EL nanodroplets specifically in the colon environment, thus achieving high-concentration treatment at the tumor lesion site (Liu et al., 2025b; Raghav et al., 2020).

**Scheme 1:**
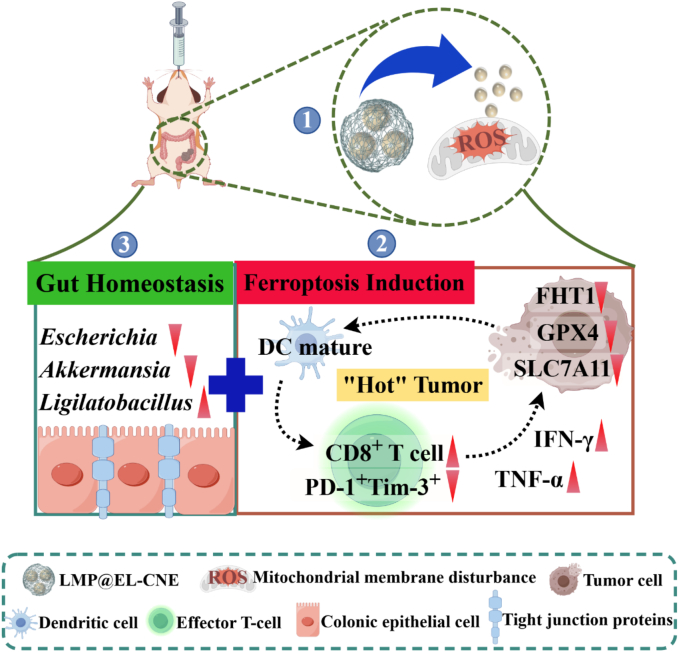
**Schematic illustration of combining ferroptosis induction and gut homeostasis restoration in colorectal cancer therapy of LMP@EL-CNE.** (1) After oral delivery, the nanoformulation disassembles in the colon *via* pH/enzyme response, releasing cationic ultra-small nanodroplets that induce mitochondrial membrane disturbance. (2) These droplets downregulate key ferroptosis regulators (including FTH1 and the SLC7A11/GPX4 axis) to trigger tumor ferroptosis, which in turn activates antitumor immunity, enhancing T-cell infiltration, elevating IFN-γ/TNF-α secretion, and establishing a “hot” tumor microenvironment with reduced PD-1/Tim-3 co-expression. (3) Simultaneously, the LMP polysaccharide armor repairs the gut barrier through tight-junction upregulation and mucus reinforcement, while selectively modulating the gut microbiota (*e.g.*, *Escherichia, Akkermansia, Lactobacillus*). These coordinated mechanisms enable a multi-strategy synergistic therapy against colorectal cancer.

Herein, we developed an LMP-armored EL nanoemulsion (LMP@EL-CNE) to address the instability of oral nanoemulsions and enable multi-strategy CRC therapy. This multifunctional armored nanoemulsion, which sequentially releases ultra-small droplets (∼20 nm), promotes EL accumulation and amplifies ferroptosis in CT26 cells. This process further activates a comprehensive anti-tumor response *in vivo*, thereby creating a “hot tumor” microenvironment. Simultaneously, it synergistically remodels gut homeostasis by repairing the intestinal barrier and modulating the gut microbiota (Scheme 1). In summary, this novel OCDDS represents a promising multi-pronged therapeutic strategy for the treatment of orthotopic CRC, effectively integrating ferroptosis induction, immunotherapy activation, and gut homeostasis restoration.

## 2 Materials and methods

### 2.1 Materials

EL and EL Oral Emulsion (EL-OE, a commercial preparation with EL Liposomes, 20 mL:196 mg) were purchased from Jingang Pharmaceutical Co., Ltd., Dalian, China. Citrus low methoxyl pectin (LMP, esterification degree 27.9%, 100 kDa) was purchased from Yuning Pectin Co., Ltd., Anhui, China. Medium-chain triglyceride Labrafac^@^WL1349 (WL1349) and diethylene glycol monoethyl ether Transcutol^@^HP (HP) were gifts from Gattefossé. Polyoxyethylene hydrogenated castor oil (RH40) and hexadecyl trimethyl ammonium bromide (CTAB) were purchased from Macklin Incorporation. β-elemene (purity>98%), simulated gastric fluid (SGF, pH = 1.3), simulated intestinal fluid (SIF, pH = 6.8), simulated colonic fluid (SCF, pH = 7.8), and pectinex®UF (≥ 3300 PGNU/g, pectinase, Pase) were purchased from Yuanye Bio-Technology Co., Ltd., Shanghai, China. D-Luciferin potassium salt, and fluorescent 1,1′-dioctadecyl-3,3,3′,3′-tetramethylindodicarbocyanine-4-chlorobenzene sulfonate (DiD), coumarin-6 (C6), and Liproxstatin-1 (Lip-1, purity >99%) were purchased from Sigma Aldrich. CM-H2DCFDA kit, BODIPY 581/591 C11kit, JC-1 kit, MDA kit, GSH kit, GSH-PX kit, mouse TNF-α kit, and mouse IFN-γ ELISA kit were purchased from Beyotime Biotechnology, Shanghai, China. All other reagents and solvents were of analytical grade.

### 2.2 Animals

BALB/C mice (male, ∼7-week-old, 18–20 g) were obtained from Hangzhou Resources Laboratory Animal Technology Company (SCXK2019–0004, Zhejiang, China). All animals were housed at 25 °C with 60–70% humidity, provided with sufficient water and food, and acclimatized for at least 1 week before the experiments. All experimental protocols were approved by the Animal Ethics Committee of the Anhui University of Chinese Medicine (AHUCM-mouse-2,024,140) and conducted in accordance with the National Institutes of Health's Principles of Laboratory Animal Care.

### 2.3 Preparation and evaluation of LMP@EL-CNE

#### 2.3.1 Preparation of LMP@EL-CNE

Firstly, EL-CNE was prepared by the self-emulsification method (Li et al., 2023). In brief, the EL-CNE formulation was determined based on our previous research, as shown in Table S1 (Supporting Information). A clear and transparent EL-CNE was obtained at 200 rpm for 5 min using a magnetic stirrer. Then, LMP@EL-CNE was prepared by the emulsion diffusion method reported earlier with slight modifications (Rosso et al., 2021; Wang et al., 2022b). Briefly, EL-CNE (125 mg) was added to 0.1% (*w*/w) LMP at a 1:3 mass ratio (Table S2), and the mixture was magnetically stirred at 100 rpm for 2 h at room temperature. Finally, centrifuged at 12000 ×*g* (Beckman, Optimax-100, USA) for 20 min at 5 °C, discard the supernatant, then washed 3 times with ultrapure water to obtain LMP@EL-CNE. For fluorescent analyses *in vitro* or *in vivo*, C6 or DiD was added to the oil phase to obtain final concentrations of 75 μg·mL^−1^ for C6 or 60 μg·mL^−1^ for DiD in the EL-free formulation (LMP@CNE), named LMP@C6-CNE or LMP@D-CNE.

#### 2.3.2 Encapsulation efficiency and drug loading of LMP@EL-CNE

The drug loading (DL%) and encapsulation efficiency (EE%) of LMP@EL-CNE were determined by High-performance liquid chromatography (HPLC; Thermo Scientific U3000, USA). LMP@EL-CNE (1 g) was dispersed in 80% aqueous methanol (50 mL) and subjected to ultrasonic for a series of three cycles, each 5 min in duration, then the supernatant was collected at 15000 ×g (Beckman, Optimax-100, USA) for 5 min. The supernatant was used as the sample solution, and calculated according to the equations:

(1) $$\;\mathrm{DL}\left(\%\right)=\left(m_{\mathrm{EL}}/m_{0}\right)\times 100\%$$

(2) $$\;\mathrm{EE}\left(\%\right)=\left(\;m_{\mathrm{EL}}/m_{1}\right)\times 100\%$$

$m_{\mathrm{EL}}$ is the total amount of the EL in the LMP@EL-CNE, and *m*_*0*_ is the total mass of the LMP@EL-CNE; $m_{1}$ is the total amount of EL added.

#### 2.3.3 The stability of LMP@EL-CNE

The stability of LMP@EL-CNE was preliminarily evaluated by examining its particle size, polydispersity index (PDI), zeta potential, and EL retention in 7 days at room temperature. Besides, LMP@EL-CNE was suspended in ultrapure water containing Pase (0.1 mM), SGF, SIF, or SCF After 1 h, the zeta potential and the particle size were recorded separately.

### 2.4 Characteristics of LMP@EL-CNE

#### 2.4.1 Morphological characteristics of LMP@EL-CNE

The morphological characteristics of LMP@EL-CNE were examined by transmission electron microscopy (TEM, Hitachi-HT7700, Japan). Its particle size, zeta potential, and PDI were determined using a zetasizer (Malvern ZS-90, UK). EL-CNE (20 mg) was added to ultrapure water (50 mL), which was magnetically stirred at 100 rpm for 5 min, and served as the control.

#### 2.4.2 Structural characteristics of LMP@EL-CNE

The thermal properties and chemical structure were assessed by differential scanning calorimetry (DSC, TA-DSC Q20 system) over a range of 20–480 °C and Fourier transform infrared (FT-IR, Nicolet iS5, USA) spectroscopy between 4000 and 500 cm^−1^, respectively, EL-CNE, and LMP were also used for structural analysis.

### 2.5 *In vitro* release characteristic of LMP@EL-CNE

#### 2.5.1 Morphological analysis during release

To visually investigate the release behavior of LMP@EL-NE in the colonic environment, LMP@EL-CNE (2 mg) was incubated with SCF containing 0.1 mM pectinase (SCF + Pase) vorticity at 37 °C (Wang et al., 2021). At specific intervals (10, 30, and 60 min), aliquots were centrifuged at 6000 ×*g*, and the supernatant was deposited onto a 200-mesh carbon-coated copper grid. After staining, the samples were imaged by TEM, and the images were processed using ImageJ.

#### 2.5.2 pH/enzyme-responsive release curve

To evaluate pH/enzyme sensitivity, dialysis bags containing LMP@EL-CNE were immersed in SGF, SIF, SCF, or SCF + Pase (Pandey et al., 2021) at 37 °C. At predetermined time points (0.5, 1.0, 2.0, 3.0, 4.0, 5.0, 6.0 h), dialysate (1 mL) was withdrawn and replaced with an equal volume of fresh pre-warmed medium to maintain a constant volume. The dialysate was sonicated in 80% aqueous methanol for 20 min, then filtered, and the HPLC was used to determine the cumulative EL release. Meanwhile, the enzymatic activity of the fecal microbiota was assessed using mouse fecal medium (MFM) from healthy mice or orthotopic tumor-transplant model mice (D'Amico et al., 2023).

### 2.6 *Ex vivo* transport studies

To investigate the GIT transit of the armored nanoemulsion, healthy mice were randomly divided into three groups (*n* = 36) and administered Free DiD, D-CNE, or LMP@D-CNE *via* oral gavage at a DiD dose of 0.1 mg/kg. At predetermined time points (2, 4, 6, 24 h), mice were anesthetized *via* inhalation of 2.0% isoflurane. Whole-body fluorescence images were acquired using an IVIS Lumina LT imaging system (PerkinElmer, USA). Subsequently, the mice were euthanized, and the entire GIT (from the stomach to the rectum) was excised. The fluorescence intensity was immediately measured and normalized to analyze DiD accumulation.

### 2.7 *In vitro* cell studies

#### 2.7.1 Cell culture

CT26.WT cells (Cobioer Biotechnology Co., Ltd.) were cultured in RPMI-1640 containing 10% (*v*/v) fetal bovine serum (FBS) and 1% (v/v) penicillin/streptomycin, and maintained at 37 °C in a humidified incubator with 5% CO_2_.

#### 2.7.2 Cell viability assay

The cytotoxicity of free EL solution, LMP@CNE, and LMP@EL-CNE was evaluated by CCK-8 kit assay. Briefly, CT26 cells were seeded at a density of 2 × 10^5^ per well in 96-well plates and cultured overnight. Subsequently, the original medium was removed and replaced with drug-containing medium at varying concentrations (0.1, 0.25, 0.5, 1.0 mM of EL) for co-incubation with the CT26 cells for an additional 24 h. PBS was used as a negative control. After treatment, the cells were rinsed with PBS, and CCK-8 solution was introduced into each well for an additional 30 min of incubation (Xiao et al., 2025). The absorbance at 450 nm was recorded. In addition, free EL solution and LMP@EL-CNE were co-incubated with ferroptosis inhibitor Liproxstatin-1 (Lip-1, 1 μM) (Zhu et al., 2025a) to verify the ferroptosis in CT26 cells.

#### 2.7.3 Cellular uptake assay

CT26 cells were seeded in 12-well plates (1 × 10^5^ cells/well) and cultured overnight. The cells were then treated with free C6 or LMP@C6-CNE (200 ng/mL of C6) in serum-free medium for 4 h (Du et al., 2023). Subsequently, the cells were thoroughly washed with PBS and counterstained with DAPI. The intracellular fluorescence, indicating cellular uptake, was observed and imaged.

#### 2.7.4 Ferroptosis induction evaluation

CT26 cells were seeded in 6-well plates at a density of 1 × 10^5^ cells per well and treated for 6 h with the following: PBS, LMP@CNE, free EL solution, LMP@EL-CNE, or LMP@EL-CNE co-incubated with 1 μM Lip-1 (LMP@EL-CNE + Lip-1). Following treatment, mitochondrial membrane potential (ΔΨm), intracellular ROS, and lipid ROS were assessed using the JC-1, CM-H2DCFDA, and BODIPY 581/591 C11 kits, respectively, according to the manufacturer's protocols. Briefly, cells were incubated with the corresponding probes, washed 3 times with PBS, and immediately imaged using a fluorescence microscope. Additionally, intracellular ROS levels were quantified by flow cytometry. The key biomarkers of ferroptosis were evaluated using commercial assay kits. The concentrations of glutathione peroxidase (GSH-PX), glutathione (GSH), and malondialdehyde (MDA) in cell lysates were measured using a microplate reader to assess antioxidant defense and lipid peroxidation levels (Sha et al., 2025).

### 2.8 *Ex vivo* bio-distribution studies

The bio-distribution of the armored nanoemulsion was investigated in CT26 orthotopic tumor-bearing mice (Xie et al., 2024). Mice were orally administered Free DiD or LMP@D-CNE (*n* = 18). At 4, 6, and 24 h, major organs (heart, liver, spleen, lungs, kidneys) and tumors were excised. The *ex vivo* fluorescence signals of the tissues were captured and quantified using the IVIS Lumina LT system and Living Image software to assess DiD accumulation and distribution.

### 2.9 *In vivo* antitumor efficacy studies

An orthotopic colorectal cancer model was established by inoculating CT26-Luc cells (purchased from Cellverse Co., Ltd., Shanghai, China) into the colonic wall of BALB/c mice. These cells stably express luciferase, enabling the monitoring of tumor growth *via* bioluminescence imaging. Model mice were randomly divided into 4 groups (*n* = 24) and treated daily *via* oral gavage for 14 days as follows: group 1 (control): PBS; group 2 (EL-free formulation): LMP@CNE; group 3: LMP@EL-CNE and group 4 (positive control): EL-OE, at a dose of 0.6 mmol/kg EL. The mice's survival was monitored daily. Body weight was measured every 3 days as an indicator of systemic toxicity. Tumor growth was quantified using the IVIS Lumina LT system on days 0, 7, 14, and 21 post-treatment initiation, following intraperitoneal injection of D-luciferin.

#### 2.9.1 Western blotting

The expression levels of ferroptosis-related proteins were detected by Western blotting (WB). Tumor tissues were homogenized on ice and centrifuged, and the protein concentration of the supernatant was determined. Subsequently, 20 μg of protein per sample was separated by electrophoresis and transferred onto a PVDF membrane. After blocking, the membrane was incubated overnight at 4 °C with primary antibodies against FTH1, GPX4, SLC7A11, and the internal control GAPDH at a dilution of 1:1000. The membrane was then incubated with an HRP-conjugated secondary antibody at room temperature. Protein signals were visualized using an ECL chemiluminescence kit, and the grayscale intensity of each band was quantified with ImageJ software.

#### 2.9.2 Flow cytometry and cytokine analysis

Tumor-infiltrating immune cells were analyzed by flow cytometry (Beckman Coulter, USA). Single-cell suspensions were prepared from tumors by mechanical dissociation and enzymatic digestion with collagenase IV. After erythrocyte lysis and washing, cells were stained with fluorescently-labeled antibodies against CD80 (PE), CD86 (APC), and CD8a (FITC). Mature dendritic cells (CD80^+^CD86^+^) and cytotoxic T lymphocytes (CD8^+^) were identified and quantified. Concurrently, TNF-α and IFN-γ concentrations in tumor lysates were determined using commercial ELISA kits according to the manufacturer's protocols.

#### 2.9.3 Histological staining

Tumor tissues harvested from the model mice were fixed, paraffin-embedded, and sectioned for histological evaluation. Hematoxylin and eosin (H&E) staining was performed for general morphological assessment. For immunofluorescence analysis, sections were deparaffinized, subjected to antigen retrieval, and incubated overnight at 4 °C with primary antibodies against GPX4 or Ki67. After washing with PBS, the sections were incubated with Alexa Fluor 594-conjugated secondary antibodies at room temperature in the dark. Nuclei were counterstained with DAPI, and images were acquired using a fluorescence microscope.

To evaluate the expression and co-localization of CD8, PD-1, and Tim-3 within the tumor microenvironment, immunofluorescence co-staining was performed on tumor tissue sections. Following deparaffinization and antigen retrieval, sections were incubated overnight with a mixture of primary antibodies: rabbit anti-CD8 (1:200), rabbit anti-PD-1 (1:200), and rabbit anti-Tim-3 (1:200). Subsequently, the sections were incubated with the corresponding secondary antibodies: Cy3-conjugated (pink, for CD8), Alexa Fluor 488-conjugated (green, for PD-1), and Alexa Fluor 594-conjugated (red, for Tim-3). Fluorescence images were captured using a laser scanning confocal microscope (LSCM, LSM900-ZEISS, Germany), and the fluorescence intensity of the target proteins in randomly selected fields was quantified using ImageJ software.

### 2.10 Safety evaluation

Heart, liver, spleen, lungs, kidneys, and colon tissues derived from model mice after 21 days were collected and fixed in 4% paraformaldehyde for histological analysis by H&E staining.

### 2.11 Gut homeostasis restoration studies

#### 2.11.1 Colonic histopathological analysis

Colon tissues were collected from three groups of mice (*n* = 9) after a 10-day treatment period: healthy controls (CON), tumor-bearing models (MOD), and LMP@EL-CNE-treated tumor-bearing mice (TRT). The tissues were fixed in 4% paraformaldehyde, embedded in paraffin, and sectioned. Tissue sections were then stained using an Alcian blue/periodic acid–Schiff (AB/PAS) staining kit and visualized under a microscope. Crypt morphology and goblet cell numbers were quantified using ImageJ software.

#### 2.11.2 Assessment of tight junction proteins

The Intestinal epithelial tight junction proteins zonula occludens-1 (ZO-1) and Occludin were studied in colonic tissue by immunohistochemistry. In brief, paraffin-embedded colon tissue sections were deparaffinized and rehydrated. After antigen retrieval in citrate buffer, sections were blocked with 5% normal serum and incubated overnight at 4 °C with primary antibodies. The following day, sections were incubated with fluorophore-conjugated secondary antibodies (Alexa Fluor 488, 594) for 1 h at room temperature in the dark. Nuclei were stained with DAPI. Slides were mounted in an anti-fade medium, and images were acquired using a laser-scanning confocal microscope. Analysis was performed with ImageJ.

#### 2.11.3 Microbiome analysis

The 16S rRNA sequencing method was used to analyze gut microbiota abundance (Wang et al., 2024). Fecal samples were collected, snap-frozen and stored at −80 °C. DNA was extracted, and 16S rRNA gene libraries (V3-V4 region) were prepared with the Illumina TruSeq Nano kit. Bioinformatics analysis was conducted in QIIME2, including ASV generation, taxonomic assignment, and alpha- and beta-diversity analyses to identify differentially abundant taxa. (Janney et al., 2020).

### 2.12 Statistical analyses

Results are expressed as mean ± standard deviation (SD) or standard error of the mean (SEM). Differences between groups were evaluated with unpaired one-way analysis of variance (ANOVA) followed by Tukey's multiple-comparison test to determine whether the differences between groups were statistically significant (* *p*-value ˂ 0.05, ** *p*-value ˂ 0.01, and *** *p*-value ˂ 0.001.

## 3 Results and Discussion

### 3.1 Preparation and characterization of LMP@EL-CNE

#### 3.1.1 Morphology, particle size, and zeta potential

**Fig. 1:**
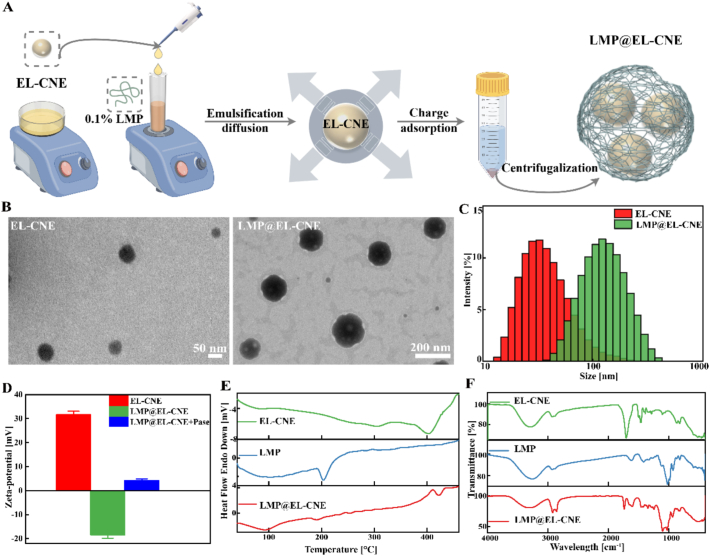
**Preparation and characterization of LMP@EL-CNE.** (A) The schematic preparation of LMP@EL-CNE. (B) Representative TEM images of EL-CNE and LMP@EL-CNE with scale bars of 50 nm and 200 nm. (C) Size distribution of EL-CNE and LMP@EL-CNE. (D) Zeta potential of EL-CNE (*n* = 3), LMP@EL-CNE, and LMP@EL-CNE + Pase. (E) DSC and (F) FT-IR analysis of EL-CNE, LMP, and LMP@EL-CNE. Data are expressed as means ± SEM.

Emulsification-diffusion plus charge-adsorption methods were used to prepare LMP@EL-CNE (Fig. 1A). The TEM images showed that the EL-CNE and LMP@EL-CNE had spherical-like morphology, and the LMP@EL-CNE with polysaccharide armor had sharper edges compared to the fuzzy edges of EL-CNE (Zhu et al., 2025b). Meanwhile, the hydration particle size of EL-CNE was 43.08 ± 2.06 nm, and that of LMP@EL-CNE was 135.60 ± 4.18 nm, further evidence that the polysaccharide-armored nanoemulsion has a more stable structure (Fig. 1B and C). The PDI of the nanoparticles was 0.191 ± 0.03 and 0.125 ± 0.02, indicating a uniform, dispersed particle size distribution. Zeta potentials of EL-CNE and LMP@EL-CNE were measured as 31.6 ± 1.47 and − 18.97 ± 0.36 mV, respectively. When incubated with Pase, the zeta potential partially reversed to 4.15 ± 0.15 mV (Fig. 1D), confirming LMP degradation and exposure of the cationic droplets (Li et al., 2025b).

#### 3.1.2 Composition and structure

To further explore the loading conditions and functional group composition of LMP@EL-CNE, DSC analysis is used to observe the mixing and dispersion of substances through thermal behaviors, indicating that components were encapsulated in LMP@EL-CNE in an amorphous form (Fig. 1E). Besides, in the FTIR spectra, the absorption peak at 1735 cm^−1^ may be related to the presence of carbonyl (C=O), and 910–950 cm^−1^ indicate to quaternary ammonium salt groups (C—N) which are the main chemical bond on the surface of EL-CNE. Meanwhile, the characteristic peak at 3550–3020 cm^−1^ of hydroxyl groups (O—H) showed broad absorption bands. The fingerprint area in the LMP was also detected as a single peak on the surface of LMP@EL-CNE, confirming that LMP was deposited on the surface of EL-CNE (Fig. 1F), consistent with the TEM images. The results further confirmed that the structure of LMP@EL-CNE provides stability and potential sustained release performance.

#### 3.1.3 Stability

The polysaccharide armor structure provided LMP@EL-CNE physical protection and pH-sensitivity (Chen et al., 2023). The EE% and DL% of LMP@EL-CNE were 79.8% ± 1.84% and 8.6% ± 0.40%, and the stability was evaluated under both storage and simulated digestive fluid conditions. First, the formulation demonstrated good short-term stability at room temperature, as evidenced by minimal changes in particle size, PDI, zeta potential, and drug retention over 7 days (Fig. S1). Furthermore, the particle size variation of LMP@EL-CNE was assessed in different simulated gastrointestinal fluids (Fig. S2). The nanoparticle maintained a monodisperse size distribution in both SGF and SIF, indicating its stability in the upper gastrointestinal tract. However, upon exposure to SCF, a broadened particle-size distribution was observed, suggesting the collapse of the LMP@EL-CNE and the subsequent release of the core.

### 3.2 Dual-responsive release and colon retention of LMP@EL-CNE

#### 3.2.1 *In vitro* dual-responsive release

**Fig. 2:**
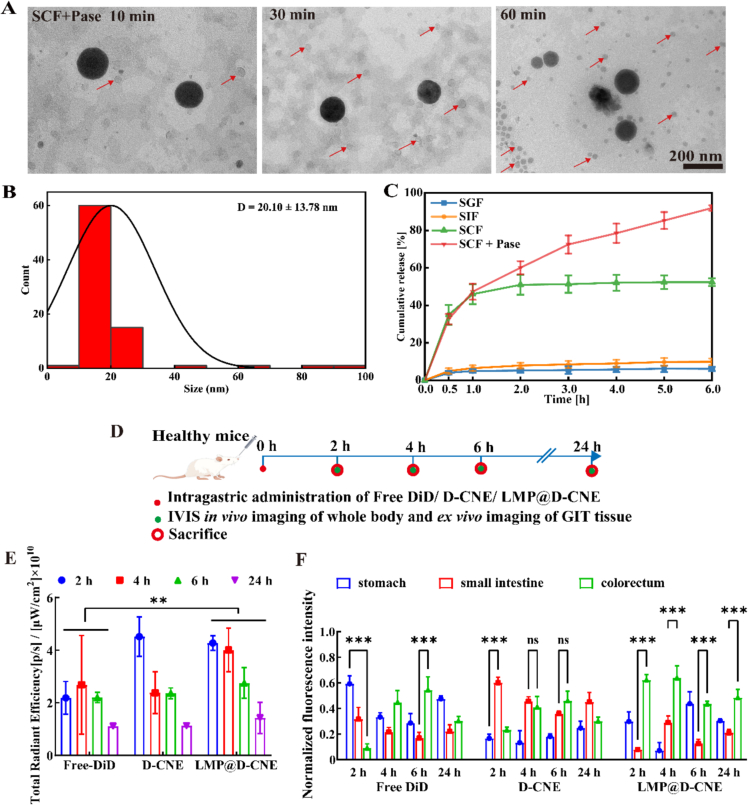
**Dual-responsive release and colon retention of LMP@EL-CNE.** (A) TEM image of the release solution incubation by SCF + Pase in 10 min, 30 min, and 60 min, scale bars 200 nm. (B) The average diameter of the LMP@EL-CNE release solution after incubation by SCF + Pase for 60 min. (C) pH/enzyme response cumulative release curve of LMP@EL-CNE in SGF, SIF, SCF, or SCF + Pase in 6 h (*n* = 3). (D) Schematic illustration of the process of gastrointestinal transit assays in healthy mice. Significance analysis of fluorescence data from (E) *in vivo* fluorescence images of the whole body and (F) *ex vivo* fluorescence imagesof gastrointestinal tissue sacrificed at 2, 4, 6, and 24 h after oral administration of free DiD, D-CNE, and LMP@D-CNE (n = 3). Data are expressed as means ± SEM; ns *p* > 0.05, ** *p* < 0.01 and *** *p* < 0.001 by one-way ANOVA with Tukey's multiple comparison test.

TEM analysis revealed the time-dependent disintegration of LMP@EL-CNE in SCF + Pase (Fig. 2A). Initially (10 min), particles remained well-dispersed but swelled to ∼200 nm. Within 30 min, sub-100 nm particles appeared; after 60 min, a homogeneous population of spherical, electron-lucent nano-droplets with relatively aggregated spatial distance appeared. The final particle size stabilizes at 20.10 ± 13.78 nm (Fig. 2B). These results demonstrate that polysaccharide armor enables a sequential release process that may enhance drug permeation for local treatment (Xi et al., 2024).

A series of simulated digestive fluids was investigated to study the dual responsiveness and bio-relevant release of LMP@EL-CNE (Sun et al., 2025a, Sun et al., 2025b) (Table S 3 and Fig. 2C). The cumulative release profile of EL revealed significant differences across media: it was stable in SGF (minimal release), showed limited release in SIF (∼ 10% at 6 h), and was progressively triggered in SCF (52.41% ± 2.19% at 6 h). Maximum release (92.01% ± 3.75% at 6 h) occurred in SCF + Pase, confirming the critical role of pH and enzymatic degradation of the polysaccharide armor (Liu et al., 2025a) (Fig. 1D). This dual-driven release was consistent in MFM (Fig. S3), confirming that the release efficacy is maintained across different physiological conditions, providing a robust foundation for *in vivo* experimentation.

#### 3.2.2 *Ex vivo* colon retention

To evaluate the GIT transit and retention of the armored nanoemulsion (Zu et al., 2024), we employed near-infrared fluorescence imaging. Healthy mice were orally administered free DiD, DiD-labeled CNE (D-CNE), or LMP@D-CNE, all possessing similar particle sizes and zeta potential (Fig. S4). The formulations were tracked at 2, 4, 6, and 24 h using an IVIS Lumina LT system (Fig. 2D), showing that the polysaccharide coating provided sustained retention, as evidenced by a significant fluorescent signal within 24 h (Table S4 and Fig. E). Moreover, *ex vivo* analysis of GIT tissues showed that LMP@D-CNE effectively redirected the distribution of the fluorescent dye from the small intestine (the primary site for D-CNE) to the colon (Table S5 and Fig. 2F). This prolonged colon residence is attributed to the synergistic effect of the polysaccharide armor's stability in the upper GIT and subsequent degradation in the colon, validating its role as an effective colon-specific drug delivery system.

### 3.3 Cell uptake and tumor targeting of LMP@EL-CNE

#### 3.3.1 *In vitro* cytotoxicity and cell uptake

**Fig. 3:**
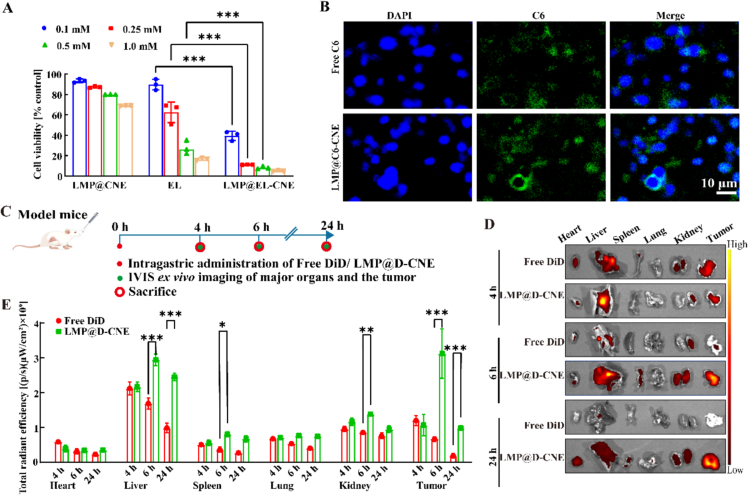
**Cell uptake and tumor targeting of LMP@EL-CNE.** (A) Comparison of cytotoxicity of LMP@EL-CNE with free EL and EL-free LMP@CNE on CT26 cells after 24 h (*n* = 6). (B) Representative fluorescence images of CT26 cells incubated with free C6 and C6-labeled LMP@C6-CNE after 4 h, with scale bars of 10 nm. (C) Schematic illustration of the process of bio-distribution assays in the CT26 orthotopic tumor model mice. (D) Representative *ex vivo* fluorescence images and (E) significance analysis of fluorescence data of heart, liver, spleen, lungs, kidneys, and tumor of mice sacrificed at 4, 6, and 24 h after oral administration of D-CNE and LMP@D-CNE (*n* = 3). Data are expressed as means ± SEM; * *p* < 0.05, ** *p* < 0.01, and *** *p* < 0.001 by one-way ANOVA with Tukey's multiple comparison test.

Nanometer-sized droplets exhibit excellent passive targeting properties, thereby achieving efficient cytotoxicity (Carvalho et al., 2022; Chen et al., 2024), as demonstrated in *in vitro* studies. When CT26 cells were treated with the solution released from LMP@EL-CNE, a significant, concentration-dependent suppression of cell viability was observed compared to an equivalent concentration of free EL solution (Fig. 3A). Meanwhile, this cytotoxicity can be reversed by a ferroptosis inhibitor, confirming that ferroptosis occurred (Fig. S5). To verify the uptake efficiency, we prepared LMP@C6-CNE with physicochemical properties similar to those of LMP@EL-CNE (Fig. S6). Confocal microscopy revealed intense green fluorescence associated with CT26 cells after treatment with LMP@C6-CNE, indicating substantial cellular uptake. In contrast, the signal from the free C6 group was markedly weaker (Fig. 3B).

The enhanced efficacy is partly attributed to the nanometer droplets formed by triggered release, which promotes cellular uptake, and partly possibly since the cationic core may facilitate its targeting of the negatively charged cancer cell plasma membrane and mitochondrial membrane, thereby inducing mitochondrial damage-a critical event in the ferroptosis pathway (Yu et al., 2022).

#### 3.3.2 *Ex vivo* tissue distribution and tumor targeting

The efficient anti-tumor activity of the armored nanoemulsion depends on its responsive release at the colon site and tumor targeting. We designed a distribution experiment in tumor-bearing mice to verify the tumor-targeting efficiency after oral administration (Fig. 3C) (Wang et al., 2022a). The fluorescence intensity analysis revealed that the LMP@D-CNE group reached its peak accumulation at 6 h, with a mean fluorescence intensity 4.4 times higher than that of Free DiD in the tumor, and showed notably high signals in the liver, spleen, and kidneys, suggesting systemic absorption and metabolism following colon delivery (Fig. 3E and Table S6). Notably, at 24 h, fluorescence remained detectable in tumor tissues from the LMP@D-CNE group (Fig. 3D), indicating sustained tumor-targeting capability of the polysaccharide-armored nanoemulsion. Based on the improved delivery efficiency of LMP@EL-CNE, our next step was to investigate whether it could exacerbate EL's inherent ferroptosis-inducing capacity and further activate the synergistic effects of chemotherapy and immunotherapy.

Although *in vitro* release data and *ex vivo* imaging results confirmed that the polysaccharide-armored nanoemulsion could release in the colon and be retained at the tumor site, we acknowledge the lack of pharmacokinetic data as a limitation of the present study. Future work will employ more sensitive detection methods, such as radiolabeling or optimized mass spectrometry, to fully characterize its pharmacokinetic profile.

### 3.4 *In vitro* ferroptosis induction of LMP@EL-CNE

**Fig. 4:**
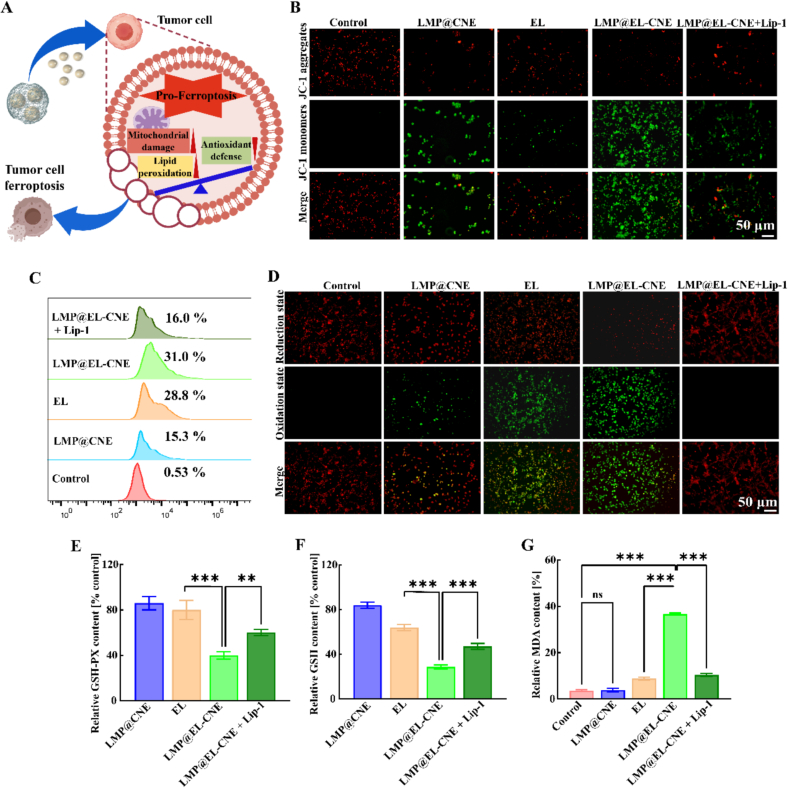
***In vitro* ferroptosis induction of LMP@EL-CNE.** (A) Schematic diagram of LMP@EL-CNE induces an imbalance in pro-ferroptosis by amplifying mitochondrial damage, downregulating antioxidant defenses, and promoting lipid peroxide accumulation. Representative fluorescence images of (B) JC-1 staining (scale bars = 50 μm), quantitative flow cytometry of (C) CM-H2DCFDA staining (*n* = 3), and representative fluorescence images of (D) BODIPY581/591-C11 staining (scale bars = 50 μm), and the quantitative accumulation of (E) GSH-PX (*n* = 6), (F) GSH (n = 6), and (G) MDA (n = 6) in CT26 cells treated by LMP@CNE, free EL, LMP@EL-CNE, and LMP@EL-CNE + Lip-1. Data are presented as mean ± SEM. Statistical significance was determined by one-way ANOVA followed by Tukey's *post hoc* test; ^ns^*p* > 0.05, ** *p* < 0.01, and *** *p* < 0.001.

EL is a broad-spectrum chemotherapeutic drug that can induce ferroptosis *via* dual mechanisms: inducing intracellular ROS (Lei et al., 2024) and inactivating GPX4-dependent antioxidant defense (Chen et al., 2020; Fu et al., 2025; Xie et al., 2020; Zhai et al., 2019; Zhao et al., 2024). This ideal positive feedback loop is often weakened by insufficient accumulation of EL within the tumor (Chen et al., 2020; Fu et al., 2025; Xie et al., 2020; Zhai et al., 2019; Zhao et al., 2024). In addition, the high mitochondrial metabolic activity of tumor cells may counteract this pro-ferroptosis effect, eventually restoring redox balance. Based on this, we hypothesize that LMP@EL-CNE acts on the mitochondrial membrane of CT-26 cells (leveraging CTAB as a common mitochondrial targeting strategy) (Nagaraj, 2025; Shi et al., 2025) to induce severe mitochondrial dysfunction. We propose that this mitochondrial dysfunction, combined with EL-induced downregulation of antioxidant defenses, synergistically disrupts the redox balance, thereby driving intense ferroptosis in CT-26 cells (Fig. 4A).

#### 3.4.1 Mitochondrial damage and ROS generation

To validate the proposed mechanism of mitochondrial dysfunction synergizing with ferroptosis induction, we first examined mitochondrial damage by measuring the ΔΨm using JC-1 probe (Fig. 4B). Compared to the control, the LMP@CNE group exhibited an increased green fluorescence (monomeric form, indicating low ΔΨm), demonstrating the intrinsic mitochondrial depolarization activity of the carrier. Furthermore, CT26 cells treated with LMP@EL-CNE showed a substantial shift from red fluorescence (aggregate form, indicating high ΔΨm) to green, suggesting severe mitochondrial damage. Conversely, in the LMP@EL-CNE + Lip-1 group, the green fluorescent monomeric form was rescued, indicating that the EL-induced, ferroptosis-associated mitochondrial damage was rescued. Then, intracellular ROS levels were further evaluated using the fluorescent probe CM-H2DCFDA (Fig. 4C). We found that EL and LMP@EL-CNE elevated ROS levels in CT26 cells to varying degrees (28.8% and 31.0%, respectively). Notably, the ROS increase induced by LMP@EL-CNE was markedly attenuated by pre-incubation with Lip-1, reducing ROS levels to 16.0%, comparable to those in the LMP@CNE group (15.3%), confirming its specificity for promoting ROS accumulation in ferroptosis induction. Consistent with fluorescence imaging of CM-H2DCFDA (Fig. S7). Interestingly, although LMP@CNE induced ΔΨm depolarization in CT26 cells, it did not trigger a substantial increase in ROS. This phenomenon may be attributed to compensatory antioxidant defenses within the tumor.

#### 3.4.2 Lipid peroxidation and antioxidant defense inhibition

To visually confirm lipid peroxidation, we used the fluorescent probe BODIPY 581/591 C11 to evaluate the degree of lipid ROS. CT26 cells treated with LMP@EL-CNE exhibited intense green fluorescence, indicating extensive oxidation of the probe and thus high levels of lipid ROS compared to LMP@CNE and EL groups. Meanwhile, the LMP@EL-CNE + Lip-1 group exhibited intense red fluorescence, indicating extensive reduction of the probe and thus reversed the increase in lipid peroxides induced by ferroptosis (Fig. 4D). Furthermore, given the unique mechanism of EL in inhibiting antioxidant defense, we examined GSH-PX and related metabolites GSH and MDA expression (Fig. 4E, F, and G). LMP@EL-CNE treatment group significantly reduced both GSH-PX levels and GSH content compared to EL groups (reduced by 50.6% in GSH-PX and 47.1% in GSH, respectively). Co-treatment with Lip-1 increased GSH-PX and GSH levels by 35.0% and 25.5%, respectively, compared to the individual LMP@EL-CNE process. Concurrently, MDA levels, an indicator of ferroptosis, were markedly elevated in the LMP@EL-CNE group, 5.3 times higher than in the EL group, and were also reversed by ferroptosis inhibitors, confirming that LMP@EL-CNE triggers a potent pro-ferroptosis effect *in vitro.*

### 3.5 *In vivo* antitumor therapy of LMP@EL-CNE

**Fig. 5:**
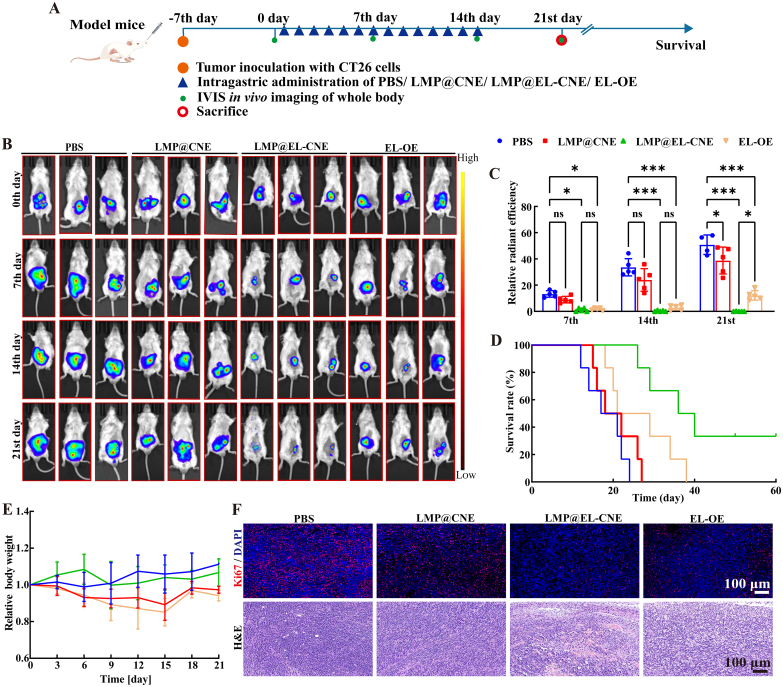
***In vivo* antitumor therapy of LMP@EL-CNE.** (A) Schematic illustration of the process of establishing, treating, and monitoring the orthotopic colorectal model. (B) Representative IVIS bioluminescence images of mice bearing orthotopic colorectal tumors over time in PBS, LMP@CNE, LMP@EL-CNE, and EL-OE groups. (C) Corresponding individual quantitative analysis of relative bioluminescence intensity on the 7th, 14th, and 21st days after different treatments. (D) Survival, and (E) Changes in body weight of mice in various groups. (F) Representative Ki67 and H&E staining of mouse tumor sections after various treatments, with scale bars of 100 μm. Data are shown as mean ± SEM (*n* = 6). Statistical significance was determined by one-way ANOVA followed by Tukey's *post hoc* test; ^ns^*p* > 0.05, * *p* < 0.05, *** *p* < 0.001.

Based on the promising *in vitro* efficacy of LMP@EL-CNE, we evaluated its therapeutic potential in an orthotopic colon cancer model. The treatment schedule spanned 14 days, with tumor progression monitored *via* bioluminescence imaging over 21 days, alongside body weight tracking and survival analysis (Fig. 5A). Bioluminescence imaging revealed that the EL-containing groups showed inhibition of tumor growth as early as 7 days after administration. At day 14, the tumors of mice in the control group were significantly enlarged, whereas the growth of tumors of mice in the LMP@EL-CNE and commercial EL-OE group was further inhibited. Notably, in treatment cessation, the tumors in the LMP@EL-CNE group continued to regress, while those in the EL-OE group began to relapse at day 21 (Fig. 5B and C). Furthermore, LMP@EL-CNE significantly prolonged survival, with a median survival time of 36.5 days and evidence of complete tumor regression in some mice. All mice in the other groups succumbed to the disease by day 38 (Fig. 5D). Crucially, this potent efficacy was achieved with minimal systemic toxicity. H&E staining of major organs (heart, liver, spleen, lungs, and kidneys) and colon tissue revealed no apparent treatment-related lesions across all groups, confirming the good biocompatibility of the LMP@EL-CNE (Fig. S8). At the same time, mice treated with LMP@EL-CNE maintained stable body weight, whereas the EL-OE group showed significant weight loss (Fig. 5E). The remarkable survival benefit, combined with the continued tumor regression after drug withdrawal, strongly suggests that LMP@EL-CNE does not merely exert a direct cytotoxic effect. It likely reinvigorates the anti-tumor immune microenvironment, thereby triggering a potent and durable adaptive immune response that controls tumor growth and prevents recurrence (Xiong et al., 2021).

### 3.6 Ferroptosis-induced immune activation by LMP@EL-CNE

#### 3.6.1 *In vivo* ferroptosis induction

**Fig. 6:**
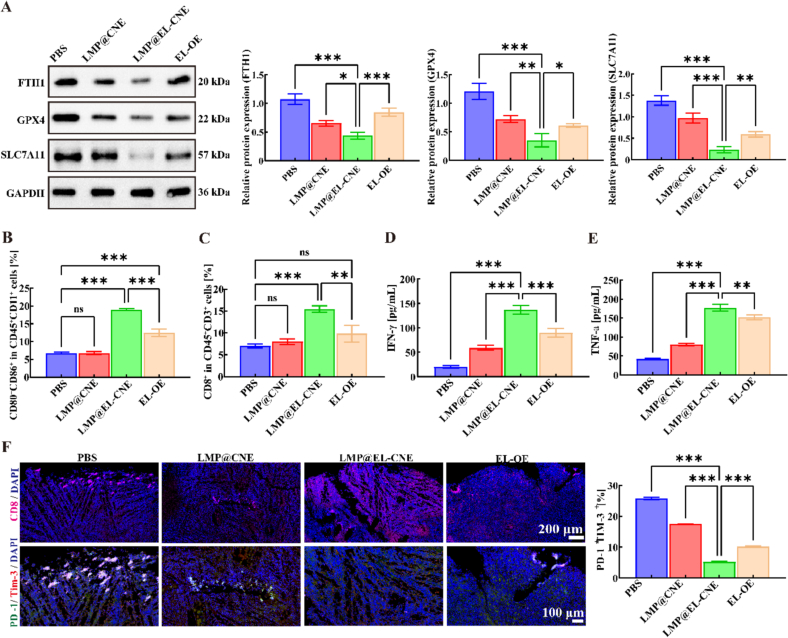
***In vivo* ferroptosis-induced immune activation by LMP@EL-CNE.** (A) WB images and quantitative analysis of FTH1, GPX4, and SLC7A11 expression, normalized to GAPDH (*n* = 3) in PBS, LMP@CNE, LMP@EL-CNE, and EL-OE groups. Quantification of mature DCs (CD80^+^CD86^+^) and (C) CD8^+^ T cells in tumor tissues by flow cytometry. (D) Concentrations of IFN-γ and (E) TNF-α in tumor tissues measured by ELISA (n = 3). (F) (Left) Representative confocal microscopy images showing CD8^+^ T cell infiltration (pink; scale bar: 200 μm) and a magnified view of the co-localization of exhaustion markers Tim-3 (red) and PD-1 (green; scale bar: 100 μm). (Right) Fluorescence quantification of the co-localization area of Tim-3 and PD-1 (*n* = 5). Data are shown as mean ± SEM. Statistical significance was determined by one-way ANOVA followed by Tukey's *post hoc* test; ^ns^*p* > 0.05, * *p* < 0.05, ** *p* < 0.01, and *** *p* < 0.001. (For interpretation of the references to colour in this figure legend, the reader is referred to the web version of this article.)

On day 21, tumor tissues from each group were excised and processed for biochemical and histopathological analyses. Ki67 and H&E staining results jointly demonstrated that the LMP@EL-CNE group exhibited the most significant anti-tumor efficacy (Fig. 5F). Ki67 staining revealed the weakest cell proliferative activity in this group. H&E staining further indicated that, compared to the other groups, tumor tissues in the LMP@EL-CNE group displayed a looser structure, significantly reduced cell density, and presented typical morphological features of ferroptosis, including vacuolization and enhanced cytoplasmic eosinophilia. To further assess *in vivo* ferroptosis induction, we utilized WB to examine the expression of key ferroptosis-related proteins FTH1, GPX4, and SLC7A11 in tumor tissues from each group. The results (Fig. 6A) indicated that critical ferroptosis defense mechanisms were inhibited. Compared to the EL-OE group, LMP@EL-CNE treatment significantly downregulated the expression of FTH1, GPX4, and SLC7A11 (by 48.8%, 41.7%, and 60.6%, respectively). This downregulation of GPX4 aligns with previously reported findings. Immunofluorescence staining of GPX4 further supported this trend (Fig. S9). Notably, the marked reduction of FTH1, a major iron-storage protein, suggests an increase in the labile iron pool. This change, potentially driven by mitochondrial dysfunction, likely exacerbates lipid peroxidation by disrupting intracellular redox balance (Jin et al., 2025a, Jin et al., 2025b; Zou et al., 2025), which is consistent with us *in vitro* observations.

#### 3.6.2 “hot” tumor microenvironment activation

Given the established role of ferroptosis in potentiating antitumor immunity, we analyzed immune cell populations and related cytokines within tumor tissues. Flow cytometry revealed that the proportion of mature dendritic cells (CD80^+^CD86^+^) in the LMP@EL-CNE group was significantly higher than in the control group and 1.5-fold greater than in the commercial EL-OE group (Fig. 6B and S10). Moreover, the intratumoral infiltration level of CD8^+^ T cells in the LMP@EL-CNE group was 2.2-fold and 1.7-fold higher than that in the control and EL-OE groups, respectively (Fig. 6C and S11). ELISA results further demonstrated that the levels of the key effector cytokines IFN-γ and TNF-α in the tumor microenvironment were significantly and synergistically elevated (Fig. 6D and E). Notably, IFN-γ secreted by activated T cells has been reported to suppress the transcription of SLC7 A11(Li and Li, 2026; Sun et al., 2025a, Sun et al., 2025b), which aligns with the marked downregulation of SLC7 A11 protein observed in this study (Fig. 6A). Based on relevant literature (Jia et al., 2026; Lan et al., 2025; Lei et al., 2024), we speculate that downregulation of SLC7A11 may render tumor cells more susceptible to ferroptosis by impairing GPX4-mediated antioxidant defense, while potentially indirectly enhancing T cell immune function. Together, these data suggest that LMP@EL-CNE may trigger a positive immunoregulatory feedback loop: induced ferroptosis activates T cells, which in turn release cytokines such as IFN-γ, further suppressing the anti-ferroptotic capacity of tumor cells, thereby creating a mutually reinforcing cycle between immune activation and ferroptosis.

Importantly, LMP@EL-CNE not only established a “hot” tumor microenvironment but also effectively suppressed T cell exhaustion. We observed sustained enhancement of CD8^+^ T cell infiltration within the tumors of this treatment group, while co-expression of the exhaustion markers PD-1 and Tim-3 was significantly reduced (Fig. 6F). This phenotype further supports the successful induction of immunoenhancing ferroptosis by LMP@EL-CNE and suggests that the polysaccharide-armored EL nanoemulsion system may possess the unique advantage of maintaining T cell function and preventing their exhaustion while activating a potent anti-tumor immune response. These findings provide new mechanistic insights for its future application in immunotherapy.

### 3.7 Gut homeostasis restoration of LMP@EL-CNE

**Fig. 7:**
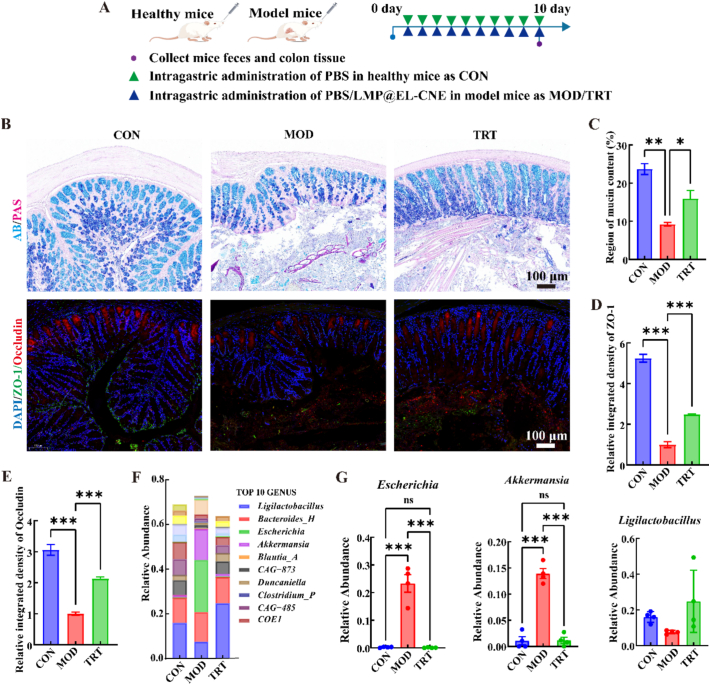
**Gut homeostasis restoration of LMP@EL-CNE.** (A) Schematic illustration of the experimental design for gut homeostasis study in the orthotopic CRC model and healthy mice. (B) Representative images of AB/PAS staining and immunofluorescence for tight junction proteins (ZO-1, Occludin) in colon tissue sections from CON, MOD, and TRT groups with scale bars of 100 μm. (C) Quantitative analysis of mucin content from AB/PAS staining. Fluorescence intensity quantification of (D) ZO-1 and (E) Occludin (*n* = 5). (F) Genus-level relative abundance of gut microbiota across groups. (G) Changes in the relative abundance of *Escherichia*, *Akkermansia*, and *Ligilactobacillus* during the 10-day treatment. Data are shown as mean ± SEM (*n* = 3). Statistical significance was determined by one-way ANOVA followed by Tukey's *post hoc* test; ^ns^*p* > 0.05, * *p* < 0.05, ** *p* < 0.01, and *** *p* < 0.001.

Impairment of the intestinal epithelial barrier is a key pathological feature of colorectal cancer (Lu et al., 2025; Pan et al., 2025). Since the colon is the primary site of action for LMP@EL-CNE, we evaluated its impact on intestinal homeostasis. Histopathological analysis of colon tissues and 16S rRNA sequencing of fecal samples were performed on healthy control (CON) mice, tumor-bearing (MOD) mice, and an LMP@EL-CNE treatment (TRT) group (10 days) (Fig. 7A).

#### 3.7.1 Repair of the intestinal epithelial barrier

AB-PAS staining revealed neatly arranged goblet cells and an intact mucus layer in the CON group, whereas the MOD group exhibited severe goblet cell atrophy and mucin depletion (Fig. 7B). The mucin-occupied area in the MOD group decreased by 61.15% compared to the CON group, while LMP@EL-CNE intervention increased the mucin area by 1.73-fold (Fig. 7C). Immunofluorescence staining further confirmed the restoration of tight junction proteins ZO-1 and Occludin to varying degrees following LMP@EL-CNE treatment (Fig. 7B). In the MOD group, ZO-1 and Occludin were likely degraded and displaced due to tumor infiltration (Chen et al., 2025) with their expression levels reduced by 80.94% and 67.32%, respectively, compared to the CON group. After LMP@EL-CNE treatment, their expression increased by 2.49-fold and 2.13-fold, respectively (Fig. 7D, E). In summary, the polysaccharide-armored nanoemulsion protected the intestinal physical barrier, which in turn may break the vicious cycle of permeability-driven CRC.

#### 3.7.2 Reverses tumor-associated gut microbiota dysbiosis

Given the established link between gut dysbiosis and colorectal cancer progression (Fong et al., 2020). we further investigated the modulatory effects of LMP@EL-CNE on the gut microbiota. The results showed that LMP@EL-CNE restored the alpha diversity of the gut microbiota in tumor-bearing mice to levels comparable to healthy controls (Fig. S12, 13). Dimensionality-reduction analysis of the species abundance matrix quantitatively illustrated differences in genus-level species composition among the groups (Fig. 5F). Finally, we identified three key genera driving these differences: *Escherichia, Akkermansia*, and *Ligilactobacillus* (Fig. 5G). i) The pro-carcinogenic genus *Escherichia*, which is associated with genotoxicity (Faghfuri and Gholizadeh, 2024; Pothuraju et al., 2021), was markedly elevated in the MOD group, while LMP@EL-CNE treatment restored its abundance to a level comparable to that in CON group. ii) *Akkermansia* (accounts for 1–4% of the gut microbiome), a mucin-degrader with a context-dependent role in CRC (Keshavarz aziziraftar et al., 2024), steering it towards a normalized, health-associated level. iii) Furthermore, LMP@EL-CNE intervention promoted an upward trend in the abundance of the beneficial probiotic *Ligilactobacillus*.

In summary, the polysaccharide armor is not merely a passive targeting component but an active modulator of gut ecology. It enables the nanoemulsion to reverse the tumor-induced and drug-aggravated barrier damage, effectively restoring a healthy-like gut microbiota composition by simultaneously suppressing pro-carcinogenic bacteria and fostering a beneficial microbial environment.

## 4 Conclusion

In conclusion, we have developed LMP@EL-CNE, an orally administered multifunctional platform that uniquely integrates ferroptosis induction, immune activation, and restoration of gut homeostasis for synergistic anti-CRC therapy. It demonstrated efficient EL stability, higher *in vitro* cell uptake, and improved cytotoxicity. The LMP shell of polysaccharide armor exhibits pH/microbial enzyme-responsive characteristics, significantly prolonging retention time in the colon and releasing EL cationic nanodroplets with an ultra-small particle size of ∼20 nm, which is the mechanism underlying increased tumor targeting. Cell line studies using CT26 cells revealed that boosting ferroptosis by increasing the imbalance in antioxidant defense. Pharmacodynamics studies on CT26 tumor-bearing BALB/c mice revealed excellent antitumor effectiveness of LMP@EL-CNE, especially with the tumor regression after drug withdrawal and a significant extension of the survival period. This can be ascribed to the high tumor accumulation, which exerts a significant ferroptosis-induced immune effect within the tumor. In addition, the regulatory role of polysaccharide armor on gut homeostasis cannot be ignored. In summary, this work establishes a promising, translatable “drug-device” strategy that highlights the immense potential of polysaccharide-based carriers for advanced oral nano-therapy in colorectal cancer.

## Date availability

Data will be made available on request.

## CRediT authorship contribution statement

**Qianyun Zhu:** Methodology, Conceptualization, Writing – original draft. **Huiru Li:** Data curation. **Wenjie Lu:** Investigation. **Dan Su:** Visualization. **Lingzhen Ding:** Validation. **Jinguang Ouyang:** Visualization. **Wenyou Fang:** Resources. **Tianming Wang:** Validation. **Shengqi Chen:** Formal analysis. **Xia Liu:** Formal analysis. **Song Gao:** Software. **Shengyong Luo:** Project administration. **Rongfeng Hu:** Supervision, Funding acquisition, Writing – review & editing.

## Declaration of competing interest

The authors declare that they have no known competing financial interests or personal relationships that could have appeared to influence the work reported in this paper.

## Data Availability

Data will be made available on request.

## Appendix A Supplementary data

Supplementary material Image 1
