# Supplementary material for: Multifunctional armored nanoemulsion of elemene combining ferroptosis induction and gut homeostasis restoration in colorectal cancer therapy
Source: Int J Pharm X. 2026 Mar 17;11:100517. doi: 10.1016/j.ijpx.2026.100517 (PMC13022687; doi:10.1016/j.ijpx.2026.100517)
Supplement: Supplementary file 1 — Supplementary material [file mmc1.docx]

# Supporting Information

Multifunctional armored nanoemulsion of elemene combining ferroptosis induction and gut homeostasis restoration in colorectal cancer therapy

Qianyun Zhu ^a,b,1^, Huiru Li ^a,1^, Wenjie Lu ^c,1^, Dan Su ^d^, Lingzhen Ding ^e^, Jinguang Ouyang ^f^, Wenyou Fang ^a^, Tianming Wang ^a^, Shengqi Chen ^a^, Xia Liu ^a^, Song Gao ^a,*^, Shengyong Luo ^b,*^, Rongfeng Hu ^a^*^,g,h,^*^*^

^a^ School of Pharmacy, Anhui University of Chinese Medicine, Hefei 230012, China.

^b^ Anhui Institute of Medicine (Anhui Academy of Medical Sciences), Hefei, 230061, China.

^c^ School of Pharmaceutical Sciences, Tsinghua University, Beijing 100084, China.

^d^ Department of Pharmacy, The First Affiliated Hospital of University of Science and Technology of China, Hefei, Anhui, 230001, China.

^e^ Anhui Zhengyao Pharmaceutical Technology Co., Ltd. Hefei, Anhui 230038, China.

^f^ Department of Gastroenterology, The First Affiliated Hospital of Anhui University of Chinese Medicine.

^g^ Anhui Province Key Laboratory of Pharmaceutical Technology and Application; Key Laboratory of Xin’an Medicine, the Ministry of Education, Anhui University of Chinese Medicine, Hefei, Anhui 230038, China.

^h^ Anhui Province Joint Key Laboratory of Functional Activity and Resource Utilization of Edible and Medicinal Mushroom, Plant active peptide function food innovative manufacturing industry innovation team, Hefei, Anhui 230038, China.

^1^ These authors contributed equally to this work.

^*^ Corresponding authors: 28552806@qq.com (S. Gao), lsy770728@126.com (S. Luo), [rongfenghu2003@hotmail.com](mailto:rongfenghu2003@hotmail.com) (R. Hu).

**Table S1.** Formulation of EL-loaded cationic nanoemulsion (EL-CNE) [%, w/w]

| Constituent | Drug | Oil | Nonionic surfactant | Co-Surfactant | Cationic surfactant |
| --- | --- | --- | --- | --- | --- |
| EL-CNE | EL | WL1349 | RH40 | HP | CTAB |
|  | 9 | 9 | 42 | 36 | 4 |

| Ratio of EL-CNE to LMP [w/w] | Size [nm] | PDI | Zeta potential [mV] | EE [%] | DL [%] |
| --- | --- | --- | --- | --- | --- |
| 1:1 | 78.82 ± 8.18 | 0.741 ± 0.21 | -8.14 ± 0.96 | 88.41 ± 1.60 | 4.62 ± 0.31 |
| 1:2 | 109.10 ± 5.02 | 0.413 ± 0.09 | -14.92 ± 0.62 | 86.32 ± 1.23 | 6.31 ± 0.44 |
| 1:3 | 135.60 ± 4.18 | 0.125 ± 0.02 | -18.97 ± 0.36 | 79.81 ± 1.84 | 8.61 ± 0.40 |
| 1:4 | 152.04 ± 3.73 | 0.312 ± 0.12 | -20.01 ± 0.53 | 64.91 ± 2.17 | 8.7 3± 0.36 |

**Table S2.** Characteristics of LMP-armored EL nanoemulsion (LMP@EL-CNE) (n = 3)

Data are expressed as means ± SD

**Table S3.** Data on the cumulative release of LMP@EL-CNE in a series of simulated digestive fluid and mice fecal medium of tumor-bearing model or healthy mice [%] (n = 3)

| Simulated digestive fluids | Time [h] | SCF | SCF (+) | Control | Model |
| --- | --- | --- | --- | --- | --- |
| SGF | 0 | 0.02 ± 0.01 | 0.04 ± 0.02 | 0.03 ± 0.01 | 0.04 ± 0.01 |
|  | 1 | 0.12 ± 0.02 | 0.14 ± 0.03 | 0.11 ± 0.02 | 0.16 ± 0.02 |
|  | 2 | 0.24 ± 0.02 | 0.22 ± 0.04 | 0.19 ± 0.03 | 0.21 ± 0.04 |
| SIF | 3 | 2.48 ± 1.57 | 2.59 ± 0.97 | 2.56 ± 1.53 | 2.45 ± 1.18 |
|  | 4 | 4.76 ± 1.52 | 4.90 ± 1.40 | 4.98 ± 1.45 | 4.67 ± 1.34 |
|  | 5 | 8.49 ± 2.25 | 8.35 ± 2.41 | 8.36 ± 1.93 | 8.19 ± 2.13 |
| SCF/MFM | 6 | 35.07 ± 5.27 | 32.62 ± 5.13 | 34.67 ± 5.14 | 26.61 ± 4.81 |
|  | 7 | 46.10 ± 5.32 | 47.33 ± 3.97 | 45.32 ± 6.05 | 39.37 ± 5.35 |
|  | 8 | 51.08 ± 5.35 | 60.15 ± 5.98 | 63.17 ± 4.95 | 53.15 ± 6.52 |
|  | 9 | 51.31 ± 4.64 | 72.52 ± 5.35 | 77.51 ± 6.30 | 66.52 ± 6.81 |
|  | 10 | 52.14 ± 4.28 | 78.51 ± 7.37 | 85.54 ± 7.36 | 73.59 ± 5.13 |
|  | 12 | 52.42 ± 3.66 | 85.32 ± 4.16 | 90.35 ± 4.63 | 80.32 ± 5.56 |
|  | 24 | 52.41 ± 2.19 | 92.01 ± 3.75 | 92.23 ± 2.79 | 89.24 ± 3.58 |

Data are expressed as means ± SD

**Table S4.** Statistical analysis of the percentages of fluorescence intensity determined by *in vivo* fluorescence images of the whole body [(p/s)(μW/cm^2^)×10^10^] (n = 3)

| Group | 2 h | 4 h | 6 h | 8 h |
| --- | --- | --- | --- | --- |
| Free DiD | 2.19 ± 0.06 | 2.68 ± 0.02 | 2.23 ± 0.02 | 1.11 ± 0.02 |
| D-CNE | 4.52 ± 0.08 | 2.38 ± 0.08 | 2.36 ± 0.02 | 1.13 ± 0.05 |
| LMP@D-CNE | 4.27 ± 0.03 | 4.01 ± 0.08 | 2.76 ± 0.06 | 1.43 ± 0.06 |

Data are expressed as means ± SD

**Table S5.** Statistical analysis of the percentages of fluorescence intensity determined by *ex vivo* fluorescence images of GIT tissue [%] (n = 3)

| Group | | Stomach | Small intestine | Colorectal |
| --- | --- | --- | --- | --- |
| Free DiD | 2 h | 0.59 ± 0.06 | 0.32 ± 0.09 | 0.09 ± 0.03 |
|  | 4 h | 0.33 ± 0.03 | 0.22± 0.03 | 0.45 ± 0.09 |
|  | 6 h | 0.29 ± 0.07 | 0.17 ± 0.04 | 0.55 ± 0.10 |
|  | 24 h | 0.48 ± 0.02 | 0.22 ± 0.05 | 0.30 ± 0.03 |
| D-CNE | 2 h | 0.17 ± 0.03 | 0.60 ± 0.04 | 0.23 ± 0.02 |
|  | 4 h | 0.13 ± 0.09 | 0.46 ± 0.03 | 0.41 ± 0.08 |
|  | 6 h | 0.18 ± 0.02 | 0.36 ± 0.02 | 0.46 ± 0.07 |
|  | 24 h | 0.25 ± 0.05 | 0.45 ± 0.07 | 0.30 ± 0.03 |
| LMP@D-CNE | 2 h | 0.30 ± 0.07 | 0.08 ± 0.01 | 0.62 ± 0.04 |
|  | 4 h | 0.07 ± 0.06 | 0.29 ± 0.05 | 0.64 ± 0.09 |
|  | 6 h | 0.44 ± 0.09 | 0.13 ± 0.03 | 0.44 ± 0.02 |
|  | 24 h | 0.30 ± 0.01 | 0.21 ± 0.02 | 0.49 ± 0.06 |

Data are expressed as means ± SD

**Table S6.** Statistical analysis of the percentages of fluorescence intensity determined by *ex vivo* fluorescence images of major organs and the tumor [(p/s)(μW/cm^2^)×10^9^] (n = 3)

| Group | | Heart | Liver | Spleen | Lung | Kidney | Tumor |
| --- | --- | --- | --- | --- | --- | --- | --- |
| Free DiD | 4 h | 0.59 ± 0.04 | 2.12 ± 0.18 | 0.51 ± 0.05 | 0.68 ± 0.03 | 0.96 ± 0.08 | 1.20 ± 0.14 |
|  | 6 h | 0.31 ± 0.07 | 1.69 ± 0.17 | 0.37 ± 0.07 | 0.54 ± 0.04 | 0.86 ± 0.02 | 0.67 ± 0.07 |
|  | 24 h | 0.23 ± 0.05 | 0.99 ± 0.14 | 0.28 ± 0.05 | 0.41 ± 0.03 | 0.76 ± 0.08 | 0.19 ± 0.07 |
| LMP@D-CNE | 4 h | 0.39 ± 0.09 | 2.17 ± 0.14 | 0.56 ± 0.08 | 0.71 ± 0.03 | 1.17 ± 0.11 | 1.07 ± 0.31 |
|  | 6 h | 0.35 ± 0.06 | 2.94± 0.17 | 0.81 ± 0.07 | 0.77 ± 0.05 | 1.38± 0.02 | 3.12 ± 0.71 |
|  | 24 h | 0.36 ± 0.06 | 2.45± 0.11 | 0.68 ± 0.09 | 0.75 ± 0.05 | 0.95 ± 0.09 | 0.98 ± 0.07 |

Data are expressed as means ± SD

**
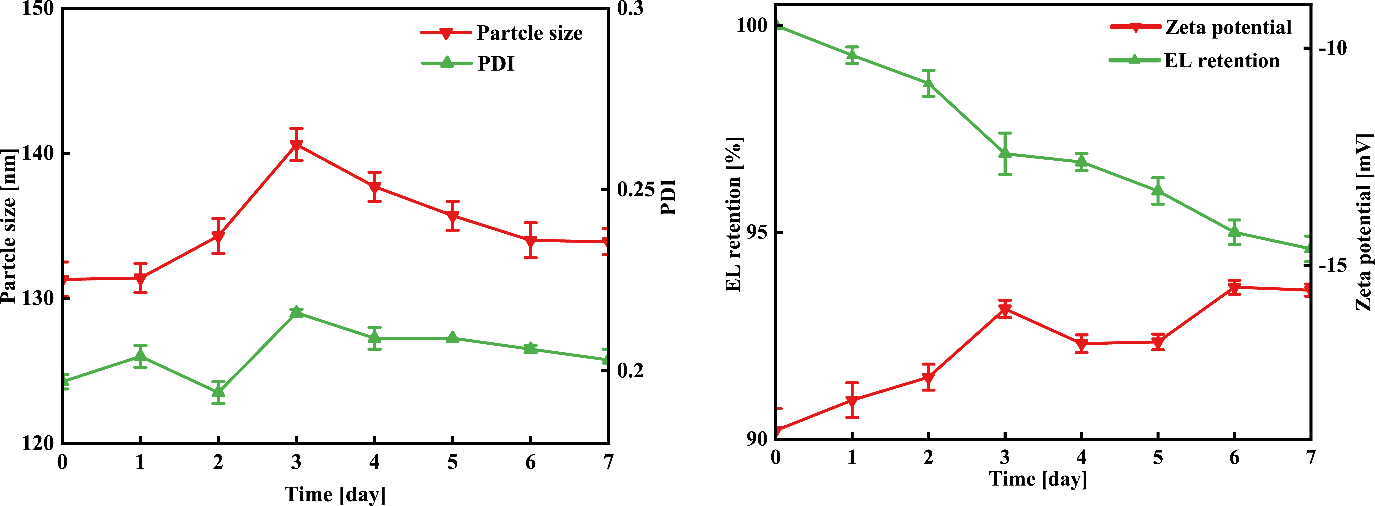
**

**Fig. S1**. Stability study of LMP@EL-CNE in 7 days by particle size, PDI, zeta potential, and EL retention. Data are expressed as means ± SEM (n = 3).

**
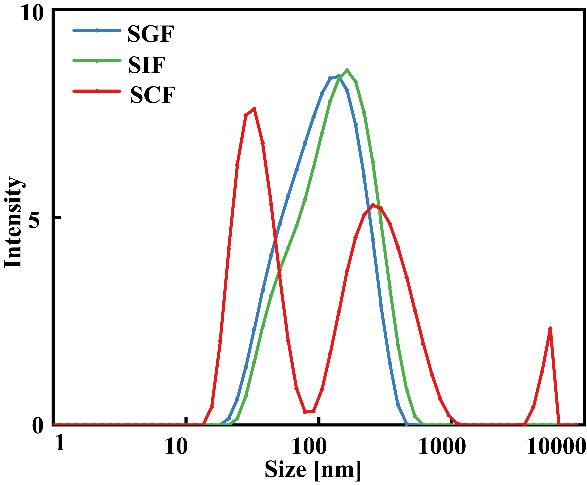
**

**Fig. S2.** The Representative particle size variation images of LMP@EL-CNE after incubation in different simulated digestive fluids (SGF, SIF, SCF) for 1 hour.


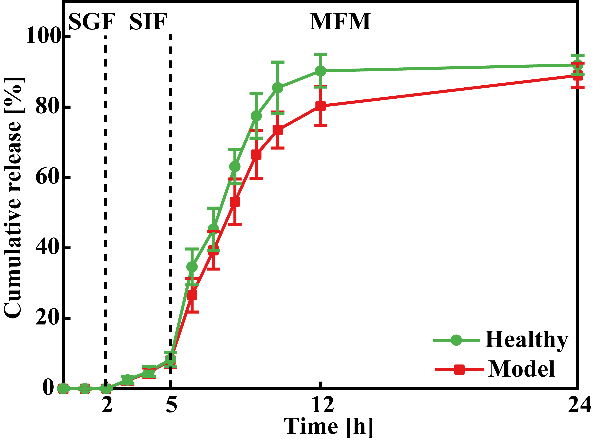


**Fig. S3.** *In vitro* bio-relevant cumulative release curve of LMP@EL-CNE in SGF for 2 hours, SIF for 5 hours, and mice fecal medium from tumor-model or healthy mice in 24 hours. Data are expressed as means ± SEM (n = 3).


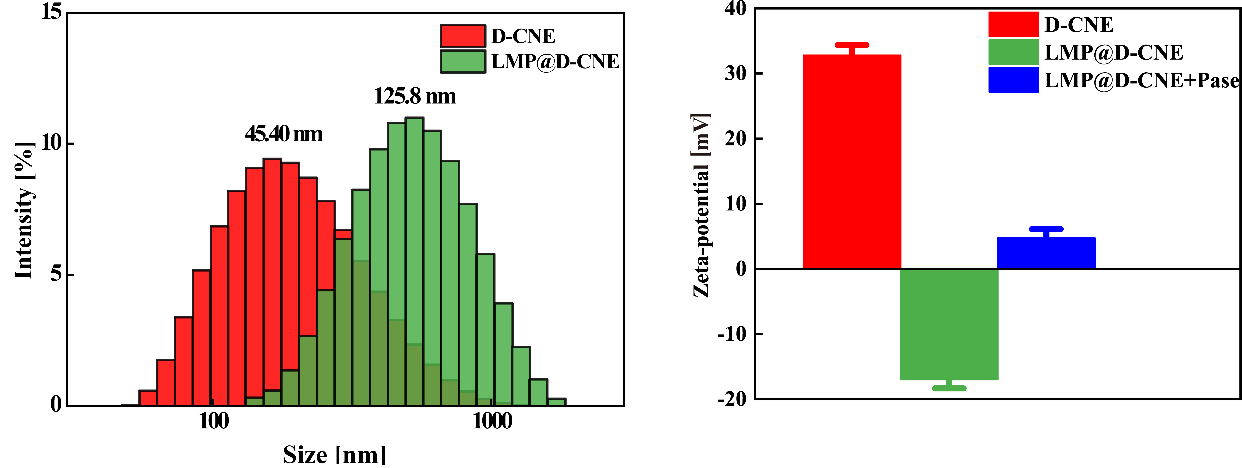


**Fig. S4.** The size distribution and zeta potential of D-CNE and LMP@D-CNE. Data are expressed as means ± SEM (n = 3).


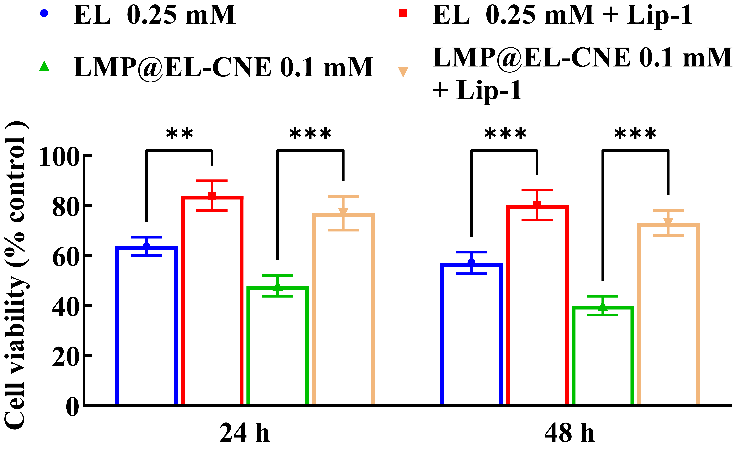


**Fig. S5.** Cell viability test with different concentrations of EL (0.1 or 0.25 mM) in Free EL, LMP@EL-CNE, and LMP@EL-CNE+Lip-1 groups for 24 h or 48 h. Data are expressed as means ± SEM (n = 6); **p < 0.01, and ***p < 0.001 by one-way ANOVA with Tukey’s multiple comparison test.

Z
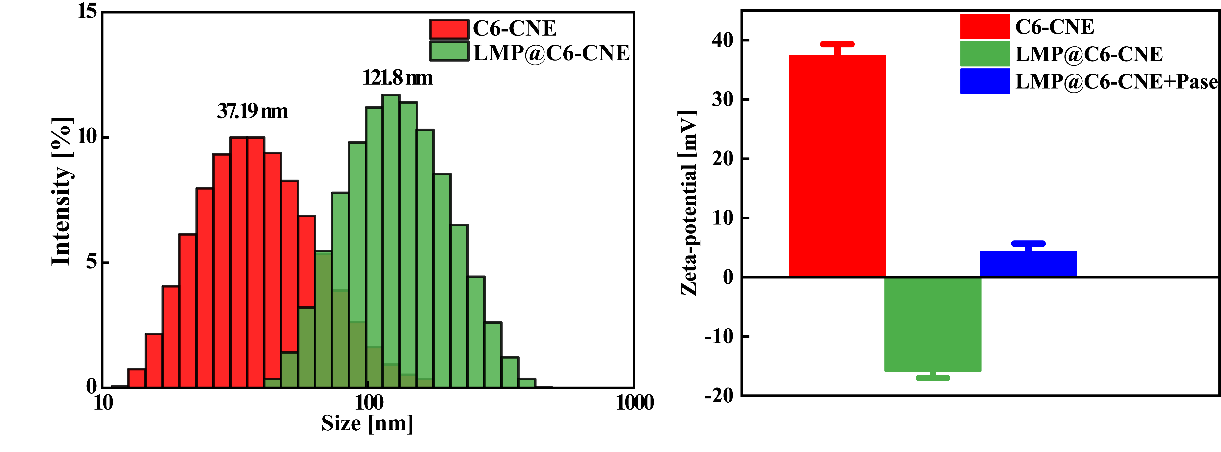


**Fig. S6**. The size distribution and zeta potential of C6-CNE and LMP@C6-CNE. Data are expressed as means ± SEM (n = 3).


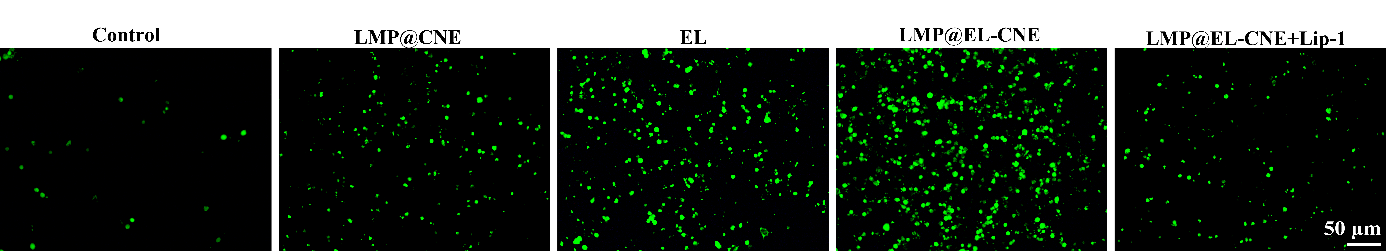


**Fig. S7.** Representative fluorescence images of CT26 cells with DCFH-DA staining in control, LMP@CNE, Free EL, LMP@EL-CNE, and LMP@EL-CNE + Lip-1 groups.


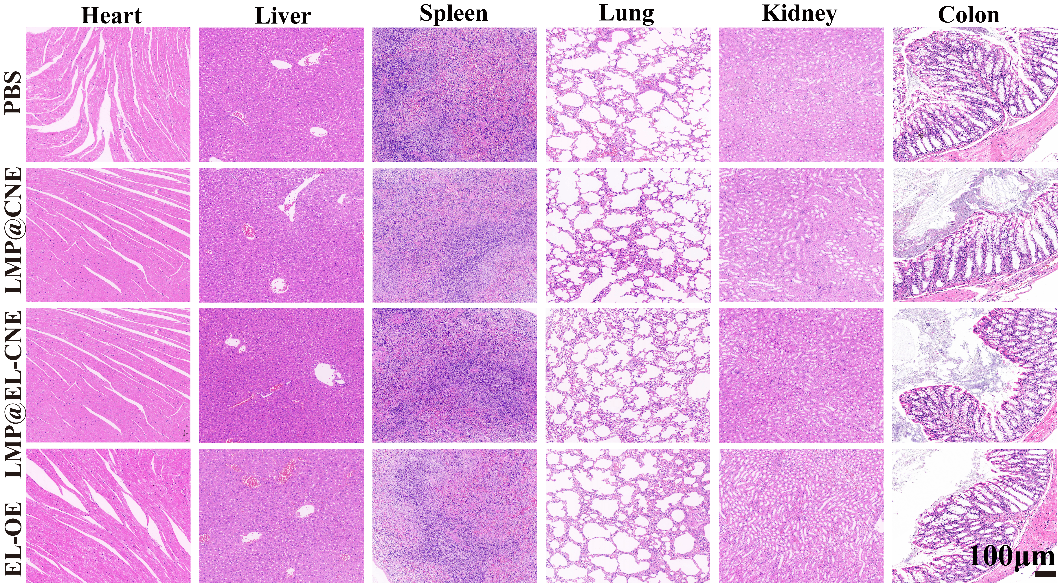


**Fig. S8.** Representative H&E staining results of mouse heart, liver, spleen, lung, kidney, and colon sections after PBS, LMP@CNE, LMP@EL-CNE, and EL-OE treatments, with scale bars of 100 μm.


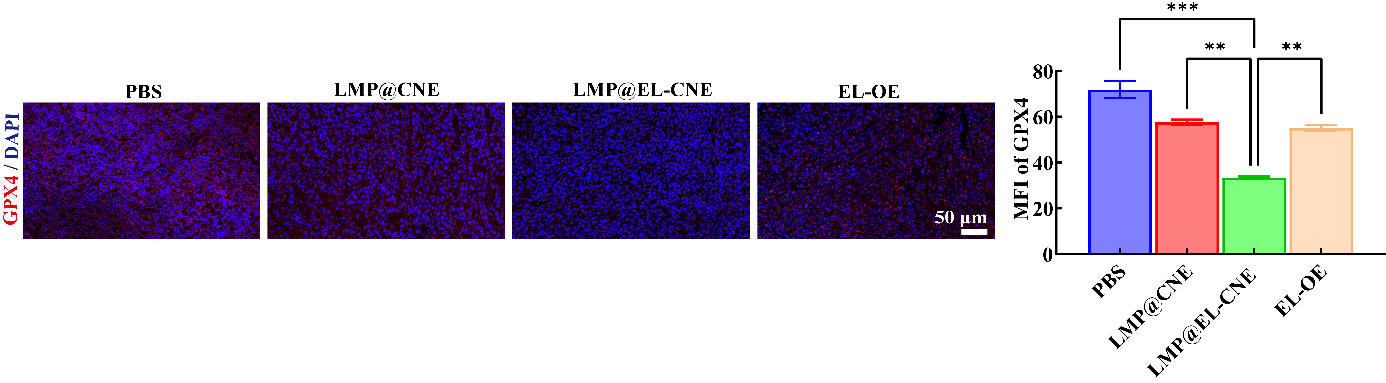
**Fig. S9.**  Representative GPX4 staining and Quantitative analysis of mouse tumor sections after PBS, LMP@CNE, LMP@EL-CNE, and EL-OE treatments, with scale bars of 50 μm. Data are expressed as means ± SEM (n = 5); ***p* < 0.01, and ****p* < 0.001 by one-way ANOVA with Tukey’s multiple comparison test.


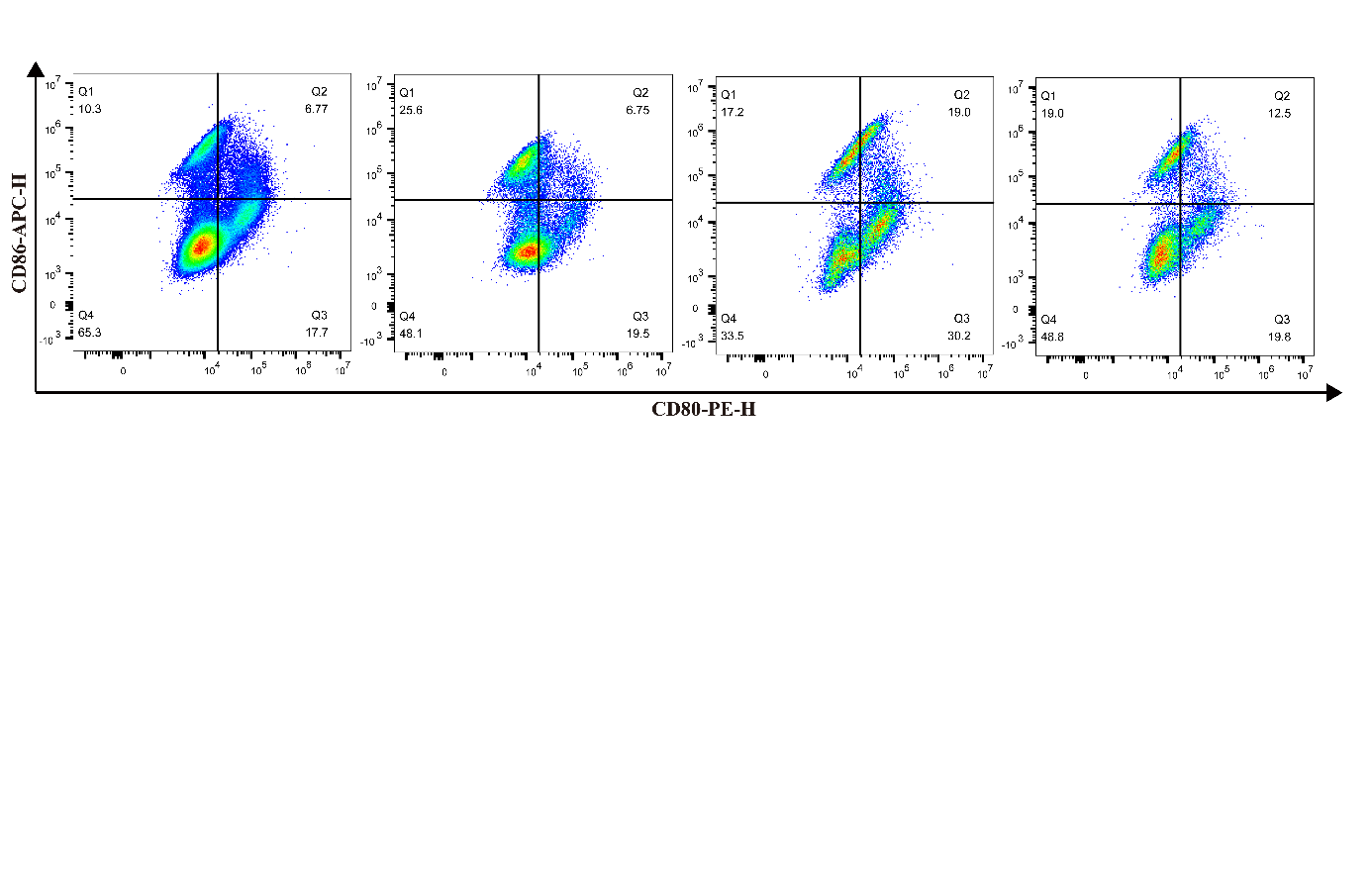


**Fig. S10.** Representative flow quantitative analysis of dendritic cells in tumors treated with PBS, LMP@CNE, LMP@EL-CNE, and EL-OE groups.


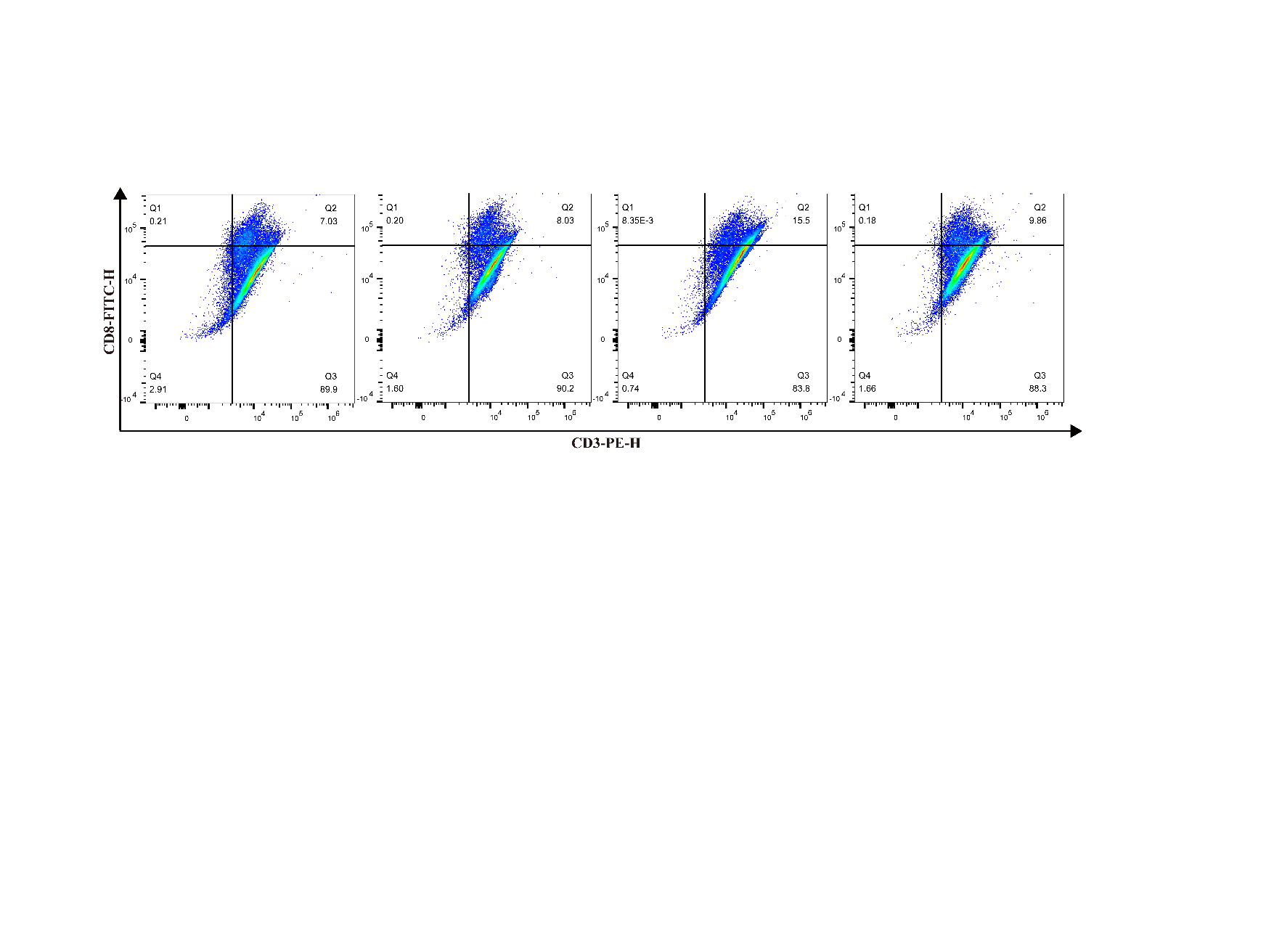


**Fig. S11.** Representative flow quantitative analysis of CD8+T cells in tumors treated with PBS, LMP@CNE, LMP@EL-CNE, and EL-OE groups.


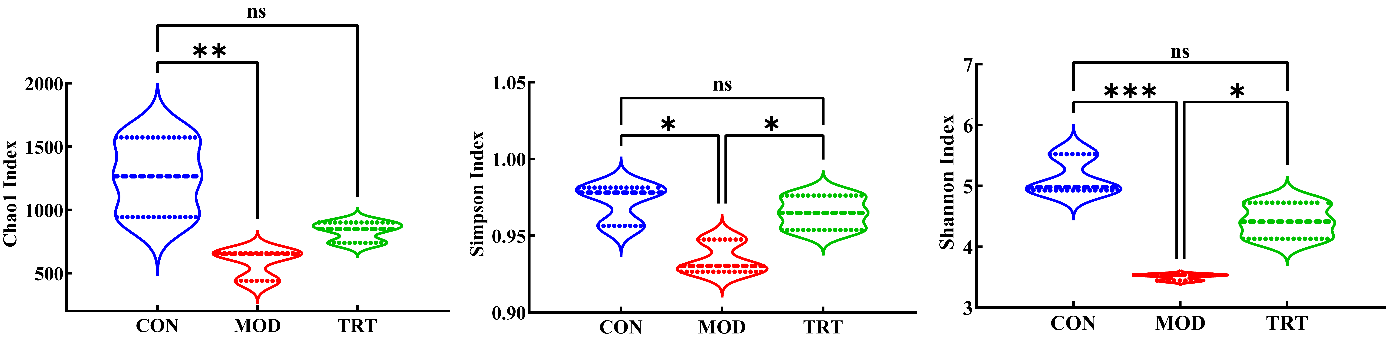


**Fig. S12.** Microbial α-diversity in terms of Chao-1, Simpson, and Shannon index at the ASV level in CON, MOD, and TRT (LMP@EL-CNE) groups, and healthy mice as the control group. Data are expressed as means ± SEM (n = 3); ns p > 0.05, **p* < 0.05, ***p* < 0.01, and ****p* < 0.001 by one-way ANOVA with Tukey’s multiple comparison test.


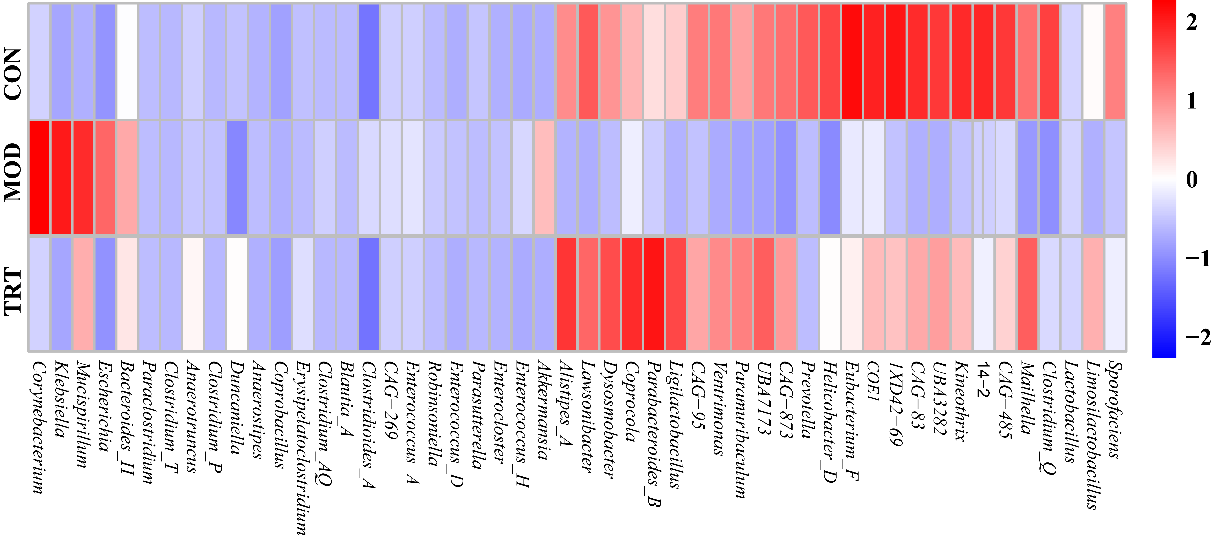


**Fig. S13.** Heatmap of horizontal species composition of CON, MOD, and TRT (LMP@EL-CNE) groups.


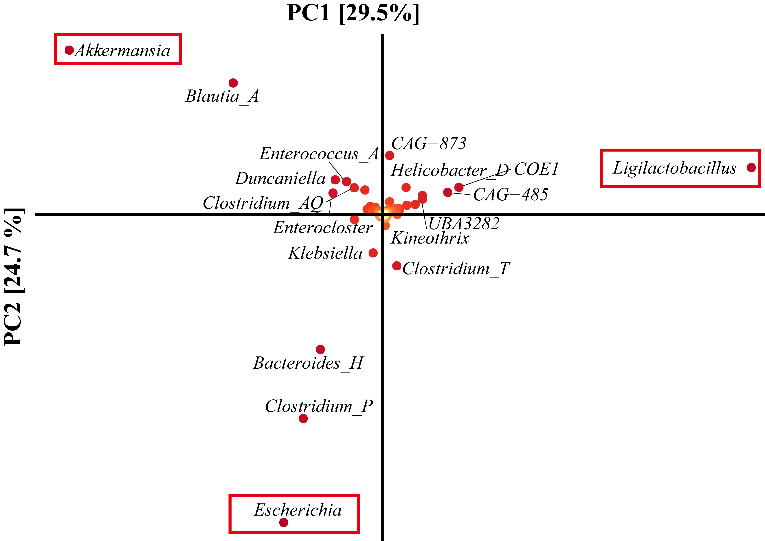


**Fig. S14.** Species loading plot for PCA analysis after various treatments.
